# A Selective Competitive Inhibitor of Aldehyde Dehydrogenase 1A3 Hinders Cancer Cell Growth, Invasiveness and Stemness In Vitro

**DOI:** 10.3390/cancers13020356

**Published:** 2021-01-19

**Authors:** Edoardo L. M. Gelardi, Giorgia Colombo, Francesca Picarazzi, Davide M. Ferraris, Andrea Mangione, Giovanni Petrarolo, Eleonora Aronica, Menico Rizzi, Mattia Mori, Concettina La Motta, Silvia Garavaglia

**Affiliations:** 1 Department of Pharmaceutical Sciences, University of Piemonte Orientale, A. Avogadro, 28100 Novara, Italy; edoardo.gelardi@uniupo.it (E.L.M.G.); giorgia.colombo@uniupo.it (G.C.); davide.ferraris@uniupo.it (D.M.F.); andrea.mangione.1@gmail.com (A.M.); menico.rizzi@uniupo.it (M.R.); 2 Dipartimento di Biotecnologie, Chimica e Farmacia, University of Siena, 53100 Siena, Italy; francesca.picarazzi@student.unisi.it (F.P.); mori.mattia@unisi.it (M.M.); 3 Department of Pharmacy, University of Pisa, 56126 Pisa, Italy; giovanni.petrarolo@phd.unipi.it (G.P.); concettina.lamotta@farm.unipi.it (C.L.M.); 4 Department of (Neuro)Pathology, Amsterdam UMC, University of Amsterdam, 1105 Amsterdam, The Netherlands; e.aronica@amsterdamumc.nl; 5 Stichting Epilepsie Instellingen Nederland (SEIN), 2103 Heemstede, The Netherlands; 6 CISUP—Centre for Instrumentation Sharing, University of Pisa, 56126 Pisa, Italy

**Keywords:** aldehyde dehydrogenases, cancer therapy, cancer stem cells, target validation, glioblastoma, biochemistry, structural biology

## Abstract

**Simple Summary:**

The aldehyde dehydrogenases enzymes (ALDHs) are promising drug targets in cancer therapy. ALDHs are members of an enzymatic superfamily composed by 19 isoforms involved in the oxidation of aldehydes, with a scavenger role. Among them, the isoform ALDH1A3 is a cancer biomarker since it is highly expressed in cancer stem cells characterized by a marked drug resistance and the capacity to promote self-renewal, clonogenic growth and tumour-initiating capacity. In this paper, we present the first highly potent and selective ALDH1A3 inhibitor able to induce cytotoxic effects and to reduce cell migration and stemness of ALDH1A3-positive cancer cells. We propose the targeting of the ALDH1A3 enzyme as a promising approach for improving the treatments outcomes of patients affected by ALDH1A3-positive cancers.

**Abstract:**

Aldehyde dehydrogenase 1A3 (ALDH1A3) belongs to an enzymatic superfamily composed by 19 different isoforms, with a scavenger role, involved in the oxidation of a plethora of aldehydes to the respective carboxylic acids, through a NAD+-dependent reaction. Previous clinical studies highlighted the high expression of ALDH1A3 in cancer stem cells (CSCs) correlated to a higher risk of cancer relapses, chemoresistance and a poor clinical outcome. We report on the structural, biochemical, and cellular characterization of NR6, a new selective ALDH1A3 inhibitor derived from an already published ALDH non-selective inhibitor with cytotoxic activity on glioblastoma and colorectal cancer cells. Crystal structure, through X-Ray analysis, showed that NR6 binds a non-conserved tyrosine residue of ALDH1A3 which drives the selectivity towards this isoform, as supported by computational binding simulations. Moreover, NR6 shows anti-metastatic activity in wound healing and invasion assays and induces the downregulation of cancer stem cell markers. Overall, our work confirms the role of ALDH1A3 as an important target in glioblastoma and colorectal cells and propose NR6 as a promising molecule for future preclinical studies.

## 1. Introduction

In humans, during numerous physiological processes, aldehydes are generated from a wide variety of precursors, including the biotransformation of most compounds such as amino acids, neurotransmitters, carbohydrates and lipids. These exogenous and endogenous compounds are tightly related with an increase of the oxidative stress and with DNA damage in cells, causing cytotoxicity and inflammation most of the time [1]. The aldehyde dehydrogenases superfamily (ALDHs) consists of 19 different isoenzymes involved in the irreversible detoxification of aldehydes to the respective carboxylic acid, using NAD(P)^+^ as a cofactor [2]. The different ALDHs enzymes act in various metabolic pathways, including detoxification of aldehydes derived from lipid peroxidation, ethanol consumption and the metabolism of several neurotransmitters [3,4]. The 19 human ALDHs protein members are divided in families in which every isoform shares at least 40% of sequence identity and in subfamilies that show 70% amino acid identity. Moreover, ALDHs have an essential role in the biosynthesis of key metabolic regulators of cellular homeostasis, such as retinoic acid (RA), γ-aminobutyric acid (GABA), dopamine and betaine [5]. In particular, the ALDH1A subfamily is composed by three different isoforms (ALDH1A1, ALDH1A2 and ALDH1A3) which are involved in the conversion of retinal, an important mediator of vision, to the retinoic acid, a potent tissue differentiation factor for cellular development [6]. Indeed, the retinoic acid signalling pathway utilizes two classes of retinoid receptors, RARs and RXRs that belong to nuclear hormone receptor family. These proteins are ligand-regulated transcription factors that bind 9-cis (RXR, RAR) or all-trans (RAR) retinoic acid via a ligand-binding domain and direct the transcription of target genes via a DNA-binding domain [7]. Several studies correlate the high ALDHs expression and their enzymatic activity to a marked chemoresistance (through the inactivation of certain chemo drugs), radio-resistance, metastasis, higher tumour invasiveness and, in general, to clinical poor outcomes [8,9,10,11,12,13]. Most importantly, the ALDHs overexpression sustains the growth of cancer stem cells (CSCs) characterized by self-renewal, clonal evolution, negatively influencing the outcome of the tumour [14,15]. In the last decade, finding new CSC markers as potential prognostic factors and druggable targets has been an important challenge, in order to develop specific cancer therapies to make the tumour less aggressive and more susceptible to specific treatments. In particular, the ALDH1A subfamily has been highlighted as a potential CSC marker in several tumour types, including brain tumours [16], colorectal cancer [17], breast cancer [18,19], hepatic cancer [20] and pancreatic cancer [21]. Increased ALDH1A subfamily activity, primary involved in RA biosynthesis associated to boosted RA receptor-mediated signalling, could be one of the most important keys in the promotion of CSCs expansion and the consequent invasiveness of the tumour [22]. One of the ALDH1A member most expressed in several cancer types is ALDH1A3. Duan et al. [23] described the overexpression of this isoform in a variety of tumours such as pancreatic cancer, ovarian cancer, and gliomas, with lower expression levels in the corresponding para-neoplastic tissues. ALDH1A3^high^ cancer cells have been described with proliferative properties and increased ability to migrate and metastasize [24]. Several small molecules targeting specific CSC signal transducer including WNT, NOTCH and HEDGEHOG have been proposed [25], and a number of them have entered Phase I of clinical trials as a strategy for targeting tumours, in which the staminal component is predominant and that have failed classical chemotherapy. In this context, may be a good strategy to target selectively ALDH1A3 to decrease the number of CSCs, and in general to guarantee a better clinical outcome. To date, the development of a selective ALDH1A3 still needs to be addressed. Different active molecules are available with different chemical scaffolds (daidizin, disulfiram) and inhibitory mechanisms, but as of today, none of these are approved for a clinical use, due to the lack of selectivity against one ALDH isoenzyme over the other members [26]. Recently, a novel class of ALDH1 inhibitors bearing the imidazo[1,2-*a*]pyridine scaffold has been described, showing significant cytotoxicity activity when tested against different glioblastoma cell lines [27,28,29]. As reported by literature, other enzyme oxidoreductase as the aldose ketose reductases (AKRs) are directly involved in the phase II of xenobiotic metabolism, with a scavenger role, and are considered as potential target for cancer therapy [30,31,32,33,34]. For example, AKR1C3 is an important oxidoreductase with multiple substrates, involved in the biosynthesis of extra-testicular androgens and it is considered as a potential target for the treatment of prostate cancer, with the aim to develop selective inhibitor [35]. Also, another isoform belonging to the same subfamily, namely AKR1C1, is known as a potential marker for the prognosis and progression of bladder cancer and also as a marker for inhibitor to improve the effects of chemotherapy [36]. In this context a significant example is AKR1B10 [37] involved also in the biosynthesis of retinol as ALDH1A isoenzymes and proposed as a target for selective inhibitor, due to its capacity to enhance the cancer cells resistance to anthracycline as the effective of the daunorubicin and idarubicin [38,39].

In this paper we present the complex between ALDH1A3 and NR6, a selective inhibitor able to inhibit selectively only one isoform, inducing cytotoxicity in ALDH1A3^high^ cells, reducing cellular motility invasiveness and stemness.

## 2. Results

### 2.1. ALDH1A3 Overexpression Is Associated with Tumour Growth in Human U87MG Glioblastoma and Human HCT116 Colorectal Cancer Cell Lines

**Figure 1:**
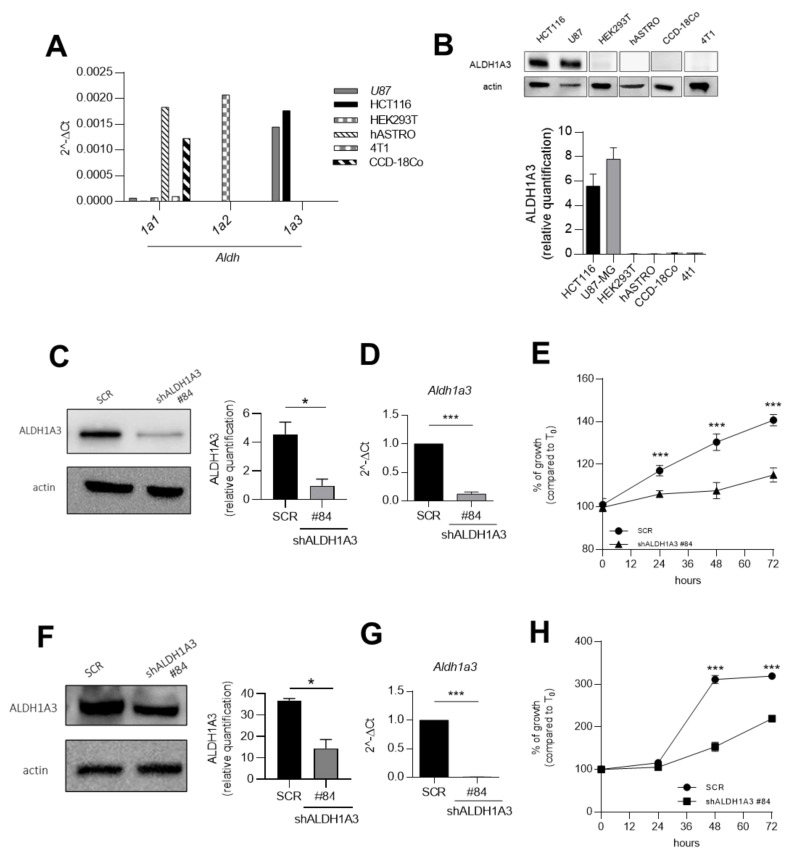
ALDH1A3 is overexpressed in glioblastoma U87MG and colorectal cancer HCT116 cell lines and its silencing determines a decrease in cell growth. (**A**) Gene expression analysis quantified by qPCR in U87MG, HCT116 cell lines and their negative controls (HEK293T, hASTRO, CCD-18Co and 4T1) of ALDH1 isoforms, *n* = 2 independent experiments. (**B**) Representative western blot (up) and quantification (down) of ALDH1A3 in U87MG, HCT116 cell lines and their negative controls. *n* = 2 independent experiments. (**C**) Representative western blot (right) and quantification (left) of shALDH1A3 knockdown of U87MG cells, *n* = 2 independent experiments. (**D**) qPCR of Aldh1a3 in U87MG cells, *n* = 2 independent experiments. (**E**) Cellular growth of shALDH1A3 U87MG cells (using shRNA, #84) compared to SCR. 4 replicates of *n* = 4 independent experiments. (**F**) Representative western blot (right) and quantification (left) of shALDH1A3 knockdown of HCT116 cells. *n* = 2 independent experiments. (**G**) qPCR of *Aldh1a3* in U87MG cells, *n* = 2 independent experiments. (**H**) Cellular growth of shALDH1A3 HCT116 cells (using shRNA, #84) compared to SCR. 4 replicates of *n* = 4 independent experiments. (*** *p* < 0.001, * *p* < 0.05 by unpaired two-tailed *t*-test).

Initially, we selected cell lines based on the expression of different ALDH1A isoforms according to The Human Protein Atlas (https://www.proteinatlas.org/). Human U87MG glioblastoma and human HCT116 colorectal cancer are labelled as ALDH1A3^+^ cell lines, HEK293T as ALDH1A2^+^ cell line, human foetal astrocytes (hASTRO) and CCD-18Co human colon fibroblasts as ALDH1A1^+^ cell line and 4T1 mammary carcinoma as triple negative ALDH1A subfamily. We checked the published data with RT-PCR as reported in Figure 1A. Our results confirm the increased expression of *aldh1a3* in U87MG and HCT116 cell lines, compared to the other cell lines. Similarly, we verify an increase of *aldh1a1* and *aldh1a2* expression in hASTRO and CCD-18Co and HEK293T, respectively. No expression of all *Aldh1a*s were detected in 4T1 mammary carcinoma cells. As shown in Figure 1B, the ALDH1A3 protein levels were evaluated with Western Blot analysis, showing its increased amount in both U87MG and HCT116 and its absence in the other cell lines selected. Consequently, we can consider both U87MG and HCT116 as ALDH1A3 positive cell lines. To better characterize the role of ALDH1A3 in these cell lines, ALDH1A3 expression was silenced. U87MG and HCT116 were transiently transfected with a shRNA (named #84, checked compared to #85, in Figure S1). The absence of ALDH1A3 expression was compared to a control shScramble (SCR) (Figure 1C,D,F–G). In order to confirm the importance of ALDH1A3 in the survival of cancer cells, we evaluated the cellular growth through MTT assay, after transfection with #84 (and #85, Figure S1). As depicted in Figure 1E, we established a decreased of 80% of U87MG cell growth at 72 h, in cells transfected with the U87MG_shALDH1A3, compared to the SCR one. Moreover, also in HCT116 cells (Figure 1H), we observed a decrease of 100% of cell growth at 72 h of HCT116_shALDH1A3 cells, compared to the control. Taken together all this data confirm the relevant role of human ALDH1A3 on cancer cell maintenance and tumour progression.

### 2.2. Functional Evaluation

The imidazo[1,2-*a*]pyridine derivative NR6 and the isomers NR2 and NR4 (Figure S2), differing from the position of the cyanide group on the pendant phenyl ring, were tested for both their inhibitory efficacy against the human recombinant ALDH1A3 and selectivity towards the ALDH1A1 and ALDH1A2 isoforms. The kinetic parameters were determined for all the compounds, following the experimental protocol described hereinafter. As shown in Figure S2, all the compounds proved to inhibit the target ALDH1A3 when tested at 25 μM concentration, displaying different degrees of efficacy towards the 1A1 and 1A2 isoenzymes. NR6, showing the best inhibitory activity at 25 μM, was investigated further to determine its IC_50_ (5.3 ± 1.5 μM) and K_i_ (3.7 ± 0.4 μM) values, which revealed a potent and competitive model of inhibition. Furthermore, the high IC_50_ and K*_i_* values of NR6 towards ALDH1A1 and ALDH1A2 confirm its high selectivity against ALDH1A3 as shown in Figures S2–S4.

### 2.3. NR6-ALDH1A3 Complex Crystallization Study

**Figure 2:**
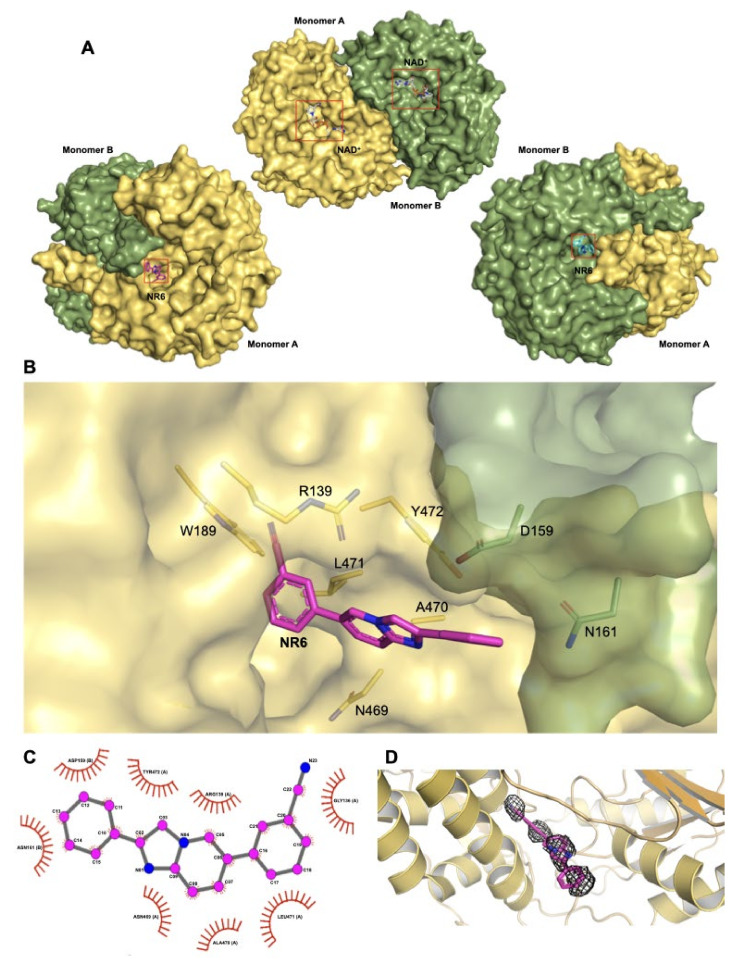
Structural analysis of ALDH1A3 in complex with NR6 (**A**) Representation of both monomer (A and B) as a surface, both are bonded with one molecule of NAD^+^ and one molecule of NR6, that are lodged with the same orientation. The inhibitor occupies only partially the entrance of the catalytic tunnel in both monomer; (**B**) Focus on the NR6 binding site: all side chains of the residues implicated in the NR6 binding are highlighted as sticks. The 2-phenyl ring and the backbone of the residues D159 and N161 from monomer B interact each other. (**C**) LigPlot+ analysis of the complex between NR6 and ALDH1A3, all the major interactions are hydrophobic, due to the nature of the molecule and the aminoacidic supply of the protein in that specific region. (**D**) NR6 2mFo-Fc map (isomesh = 0.8 carve = 1.6).

**Figure 3:**
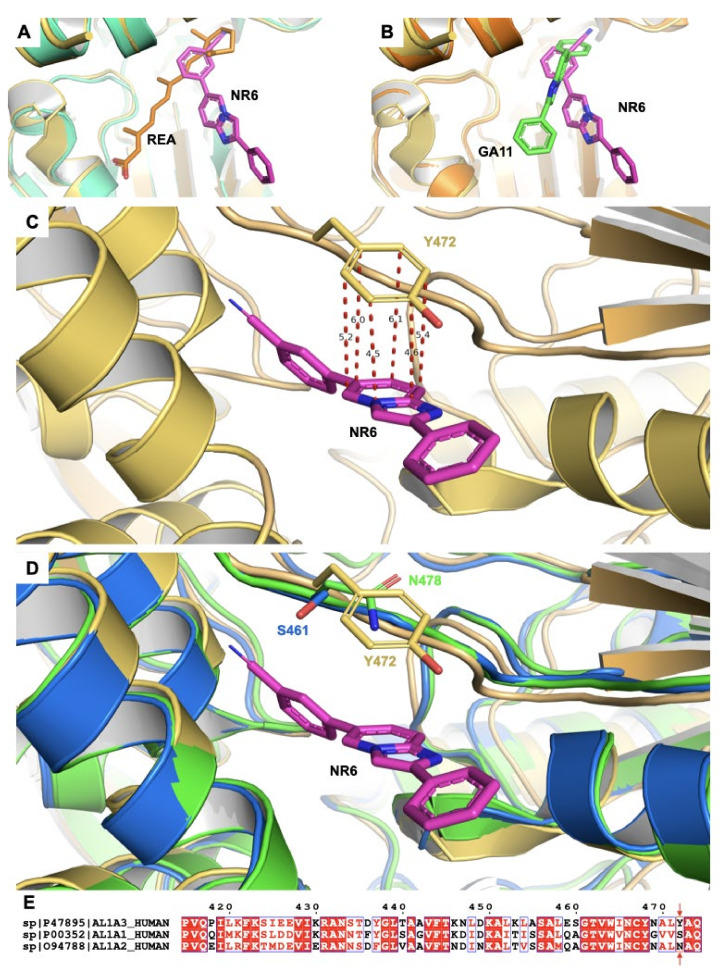
Structural superimposition between the complex ALDH1A3-NR6, other ALDH1A3 complexes (PDB 5FHZ and PDB 6S6W and the ALDH1A1 (PDB 4WB9) and ALDH1A2 (6B5G) to describe the key residue for the selectivity (**A**) Superimposition between monomers A from PDB 5FHZ (turquoise) and monomer A from PDB 7A6Q (yellow), with a focus on the binding region of the retinoic acid (REA) in the open conformation, shared with NR6. In particular, the 6-phenyl ring of NR6 ring conserved the main interactions as the cyclohexene ring of the natural product. (**B**) Superimposition between monomers A from PDB code 6S6W (orange) and monomer A from PDB code 7A6Q (yellow), highlighting the common pose at level of the 6-phenyl ring while the heterocycle is shifted to another region always at the entrance of the catalytic site (the main interaction of GA11 is with F131). (**C**) Focus on the hydrophobic interaction mediated by the tyrosine and the pyridine ring of the heterocycle. The distances between all the single atoms participating to the edge-to-face π-π stacking between the Y472 from ALDH1A3 and NR6 are represented in red. (**D**) Superimposition between ALDH1A3 (yellow, PDB code 7A6Q), ALDH1A1 (blue, PDB code 4WB9) and ALDH1A2 (green, PDB code 6B5G), the tyrosine is not conserved in the other two isoforms. (**E**) Sequence alignment between the three isoforms, a single residue seems to give high selectivity to NR6 against ALDH1A3 toward the other members of the subfamily.

The data shown highlight the importance of ALDH1A3 in the cell viability and NR6 appears to be a promising selective inhibitor. The development of a selective ALDH inhibitor is a difficult challenge, especially when a selective inhibition for the 1A1 and 1A2 isoenzymes is also pursued. As detailed in the experimental procedures, we co-crystallized ALDH1A3 and NR6 and solved the complex structure at a final resolution of 2.9 Å. The final model contains two identical chains per asymmetric unit, arranged as dimer and a total of 38 solvent molecules (Table S1). Our crystallographic data revealed that, in all monomers, NR6 binds to the enzyme at the entrance of the catalytic tunnel (Figure 2A), and no direct interactions between the catalytic cysteine and the ligand are present. The inspection of the electron density map of the NR6-ALDH1A3 complex revealing NR6 binding pose is depicted in Figure 2B. The NR6 heterocyclic cores lie in a conserved hydrophobic pocket located at the entrance of the catalytic tunnel. In this conformation, the phenyl ring in position 6 of the nucleus establishes contacts with the protein backbone, with Van der Waals contacts and hydrophobic interactions with the residues G136, R139, W189, N469, A470, L471 and Y472 (Figure 2B,C). It is worth noting that this is the same binding-site occupied by the cyclohexene ring of the natural ligand retinoic acid, by phenyl group of GA11 and its derivatives, how recently highlighted in previous published data [27,28,40], (Figure 3A). All mentioned amino acids, excluded the Y472, are conserved in each of the 1A1, 1A2, and 1A3 isozymes, highlighting this enzymatic area as “stabilizing ligand binding site” in the whole members of the 1A subfamily. A structure comparison between hALDH1A3 in complex with the progenitor of the imidazo[1,2-*a*]pyridine series, namely GA11, and NR6, provides interesting suggestions. GA11, being devoid of any substituents on the phenyl rings in position 6 of the heterocycle core, is a symmetric compound that can bind the macromolecule with two different poses, while its best derivative NR6 is able to bind ALDH1A3 with only one conserved pose. Indeed, the cyanide group blocks the inhibitor in a rigid and conserved pose in both the monomers (Figure 3B). As clearly shown in Figure 3C, the Y472 strongly binds and coordinates the pyridinic ring of NR6 with an edge-to-face π-π stacking interaction. All these interactions are further stabilized by a hydrogen bond that is established between the -OH group of the Y472 and the N1 of NR6 (3.7 Å). It is important to highlight that Y472 residue is not conserved in the ALDH1A1 and in the ALDH1A2, where it is substituted with a S461 and N478, respectively. Although ALDH1A3 shares 71% and 72% amino acid identity with human ALDH1A1 and ALDH1A2 isoenzymes, respectively, the Y472, essential in the coordination with the inhibitor, differentiates the binding pocket of the target ALDH1A3 from the one of the parent isoenzymes (Figure 3D and Figure S4). These two amino acids would retain the hydrophobic interactions unchanged, but the strong coordination with the aromatic group become completely lost. Indeed, GA11 could interact with Y472, but with a less affinity and low efficiency than NR6. Actually, the presence of the cyanide group promotes the distinct orientation of NR6 respect to Y472, thus stabilizing the binding with the protein and determining high selectivity towards ALDH1A3 (Figure 3D,E).

### 2.4. Molecular Dynamics Study of the Mutant ALDH1A3 Y472A in Complex with NR6

**Figure 4:**
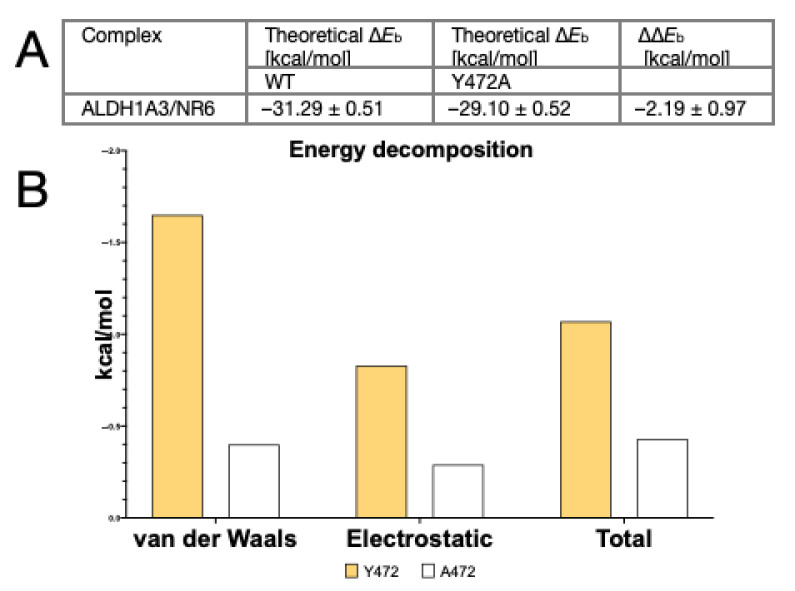
(**A**) Table of MM-GBSA-Alanine scanning theoretical affinity of ALDH1A3/NR6 complex. (**B**) Energy contribution of residue 472 to the predicted theoretical affinity of NR6 in the WT (yellow) and Y472A mutant (white) complexes.

To gain further insights into the role of Y472 in the binding of NR6 near the entrance of ALDH1A3 catalytic tunnel, molecular dynamics (MD) simulation was carried out starting from the crystallographic complex. The specific contribution of Y472 to NR6 theoretical affinity for ALDH1A3 was estimated by computational alanine scanning [41], while the delta energy of ligand binding was computed using the Molecular Mechanics Generalized Born Surface Area (MM-GBSA) approach [42]. Alanine scanning calculations allowed to quantify the role of a specific amino acid in a protein/ligand complex as the difference (delta-delta energy of binding ∆∆*E*b) between the ligand’s theoretical affinity for the wild-type (WT) sequence and that for the alanine mutant (Y472A in our case). Results showed that Y472 strongly contributes to the theoretical affinity of NR6 for ALDH1A3 with a ∆∆*E*b of −2.19 kcal/mol (Figure 4A). To deeply investigate the nature of the energy contribution provided by Y472, the theoretical affinity was then decomposed in van der Waals and electrostatic terms. From these results, it clearly emerges that the greatest energetic contribution of Y472 compared to the A472 is attributable to van der Waals interactions (Figure 4B), in agreement with the π-π stacking interaction observed between Y472 and NR6 in the crystallographic structure.

### 2.5. ALDH1A3 Inhibition Triggers ALDH1A3^+^ Cell Death

**Figure 5:**
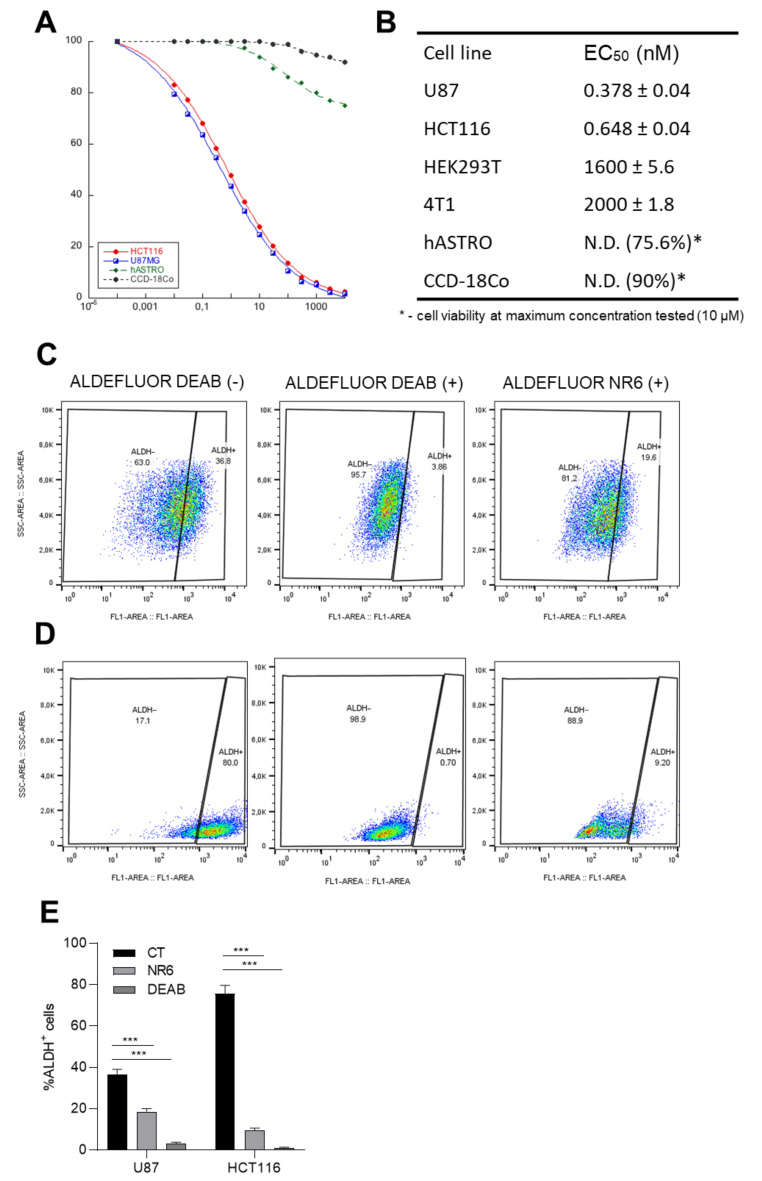
ALDH1A3 inhibitor NR6 determines cell death in ALDH1A3-overexpressing cells, reducing ALDEFLUOR positivity. (**A**) Dose-response curves of U87MG, HCT116, hASTRO and CCD-18C_O_ cell lines treated with NR6 for 72 h. (**B**) EC_50_ table values. EC_50_s were calculated with concentration–response curves and using Kaleidagraph software. Shown are mean percentages ± SD of three independent experiments (*n* = 12 for viability). (**C**,**D**) ALDEFLUOR assay in U87MG and HCT116 respectively, treated with ALDEFLUOR probe only (on the left), 10 μM DEAB as positive control (in the center) and with 1 μM NR6 (on the right). (**E**) Percentage of ALDH positive cells evaluated with flow cytometer, 2 replicates of *n* = 4 independent experiments. (*** *p* < 0.001 by unpaired two-tailed ANOVA test).

Understanding the ALDH1A3 essentiality on U87MG and HCT116 viability, NR6 was tested on ALDH1A3^+^ cell lines through a dose-response curve, using MTT assay, comparing the two cell lines to HEK293T (ALDH1A2-expressed), hASTRO and CCD-18Co (ALDH1A1-expressed) and 4T1 (not expressing ALDH1A subfamily). Figure 5A clearly shows that after 72 h-treatment, we can significantly appreciate a high picomolar EC_50_ for U87MG and HCT116 cells, 0.378 ± 0.04 nM and 0.648 ± 0.04 nM, respectively, while ALDH1A3 negative cell lines do not reveal to be susceptible to NR6 (Figure 5B). Indeed, HEK293T and 4T1 cell lines show a micromolar EC_50_ at 72 h-treatment, while hASTRO and CCD-18Co show only the 75.6% and 90% of viability at 10 μM concentration after 72 h of treatment, respectively. We assumed that also in this in vitro context, NR6 is strictly selective against the ALDH1A3 isoform in both U87MG and HCT116 cell lines, inducing apoptotic death (Figure S5). To confirm the direct impact of NR6 on ALDH1A3 subfamily activity, we performed an ALDEFLUOR assay, a no-selective probe for some ALDH isoforms [43], that it is able to detect ALDHs activity through flow cytometry analysis (gating strategy in Figure S6). As reported in Figure 5C, we performed ALDEFLUOR assay on both U87MG cells and on HCT116 cells, as well as on ALDH1A3 negative cells HEK293, hASTRO and 4T1 (Figure S7). As expected, the ALDH positive cell numbers decreased with 10 μM DEAB treatment, a pan ALDH inhibitor. Notably, 30 min-treatment of 1 μM of NR6 determined a significant decrease of ALDH-positive cells in both U87MG and HCT116. The reduction determined by NR6 treatment is associated with the ability to be more selective to one isoform, as values are higher than DEAB-treatment, and it is able to counteract the activity of other ALDH isoforms, determining a more drastic reduction of ALDEFLUOR positivity. The number of ALDH positive cells were quantified as shown in Figure 5E, observing a 20% reduction of ALDEFLUOR positivity in U87MG and a 70% reduction in HCT116 cell line, treated with NR6 (Figure 5C,D).

### 2.6. NR6 Is Able to Reduce Migration, Invasiveness and Stemness of Human U87MG Glioblastoma and Human HCT116 Colorectal Cancer Cell Lines

**Figure 6:**
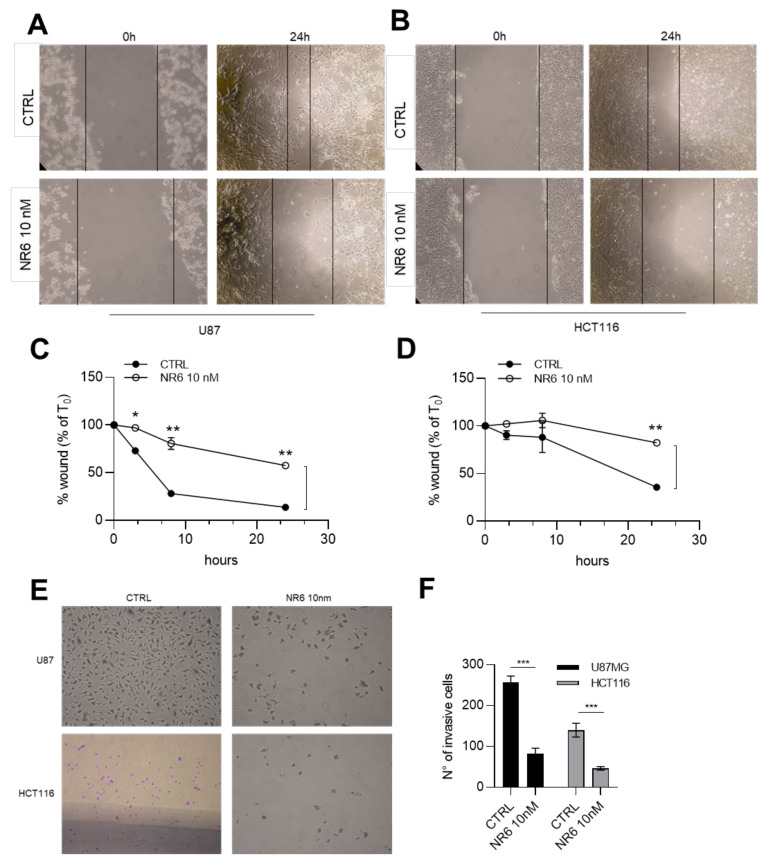
ALDH1A3 inhibitor is able to reduce migration and invasiveness in vitro. (**A**) Representative wound healing images of U87MG cells treated with vehicle and NR6 10 nM. (**B**) Representative wound healing images of HCT116 cells treated with vehicle and NR6 10 nM. (**C**) Percentage of U87MG wound closure (compared to % of the control) after 24 h of treatment. (**D**) Percentage of HCT116 wound closure (compared to % of the control) after 24 h of treatment. (**E**) Representative images of invasive assay of U87MG and HCT116 cells treated with the control and NR6 10 nM. (**F**) Number of invasive cells. Two replicates of *n* = 4 independent experiments. (*** *p* < 0.001, ** *p* < 0.01, * *p* < 0.05 by unpaired two-tailed *t*-test).

**Figure 7:**
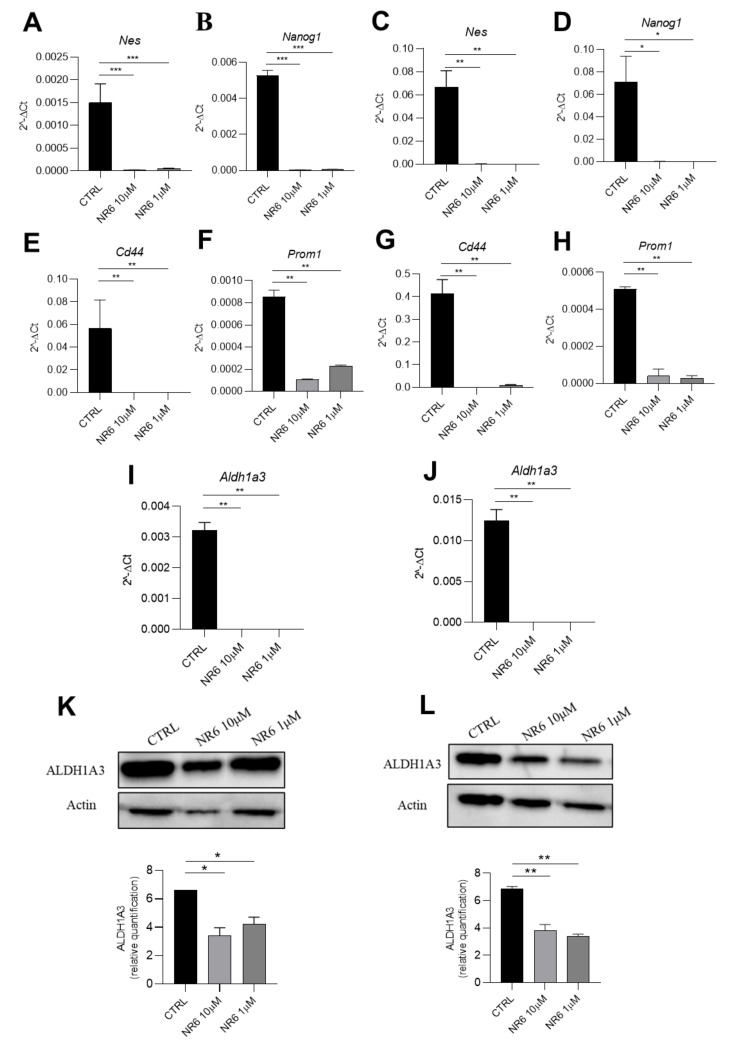
ALDH1A3 inhibitor is able to reduce stemness in vitro. Gene expression analysis quantified by qPCR of (**A**) Nes; (**B**) Nanog1 in HCT116; (**C**) Nes; (**D**) Nanog1 in U87MG cells. Gene expression analysis quantified by qPCR of (**E**) Cd44; (**F**) Prom1 in HCT116 and (**G**) Cd44; (**H**) Prom1 in U87MG. Gene expression analysis quantified by qPCR of Aldh1a3 (**I**) in HCT116 and (**J**) U87MG cells. Cells were treated with vehicle, NR6 10 and 1 μM. (**K**) Representative western blot (top) and quantification (bottom) of shALDH1A3 in HCT116 cells treated with vehicle, NR6 10 and 1 μM. (**L**) Representative western blot (top) and quantification (bottom) of shALDH1A3 in U87MG cells treated with vehicle, NR6 10 and 1 μM. Values are means ± SEM (*** *p* < 0.001, ** *p* < 0.01, * *p* < 0.05, with non-parametric Kruskal-Wallis test followed by a Dunn’s multiple comparison test).

Once assessed that ALDH1A3 is fundamental for U87MG and HCT116 migratory ability (Figure S10), we next investigated whether ALDH1A3 inhibitor NR6 may interfere with the metastatization potential, using two different assays able to determine migratory ability and invasiveness of U87MG and HCT116. These assays were performed using a 10 nM of NR6, a no-cytotoxic concentration at 24 h (Figure S8). First, we performed wound healing assay. As shown in Figure 6A,B, NR6 (10 nM) was able to reduce wound closure, evaluating the percentage of the wound (compared to the respective T_0_) of 60% for U87MG, against the 10% of the control, and of 87% for HCT116 compared to 35% of the control (Figure 6C,D), while in 4T1 cells (ALDH1A negative cells) NR6 was not able to counteract with the migratory ability (Figure S9). Moreover, to better highlight the invasive properties, a cell invasion assay was performed. We observed that after 24 h, a number of cells, for both U87MG and HCT116, are able to freely migrate from one side of a Transwell chamber to the other side (Figure 6E), but after 24 h-treatment of NR6 10 nM, the number of invasive cells is significantly reduced (Figure 6F). With these results, we demonstrate that ALDH1A3 inhibitor NR6 is able not only to interfere with cell growth, but also with the migratory and invasive potential of U87MG and HCT116 cancer cell lines. To investigate the mechanism by which NR6 exerts its anti-tumorigenic and anti-metastatic effects, we evaluated expression of gene markers associated with CSCs upon ALDH1A3 inhibition treatment. First of all, as highlighted in Figure S10, ALDH1A3 is essential for CSC preservation. Thus, we checked the expression of *Nes* and *Nanog1* as general stem cell markers, and their reduction after NR6 treatment at 10 μM and 1 μM is significant compared to the control at 4 h, as shown in Figure 7A–D. Moreover, in presence of NR6, in both U87MG and HCT116, we demonstrate a decrease of the expression of *Cd44* and *Prom1* (Figure 7E–H). Importantly, we want to highlight the significance of the gene expression reduction of *Cd44* in U87MG [16]. ALDH1A3 has been emphasized as mesenchymal marker in glioblastoma cells. As reported in Figure 7G, *Cd44* is highly downregulated upon NR6 treatment, as another evidence of ALDH1A3 involvement in CSC functionality. In conclusion, for both U87MG and HCT116 cell lines, a reduction of CSC markers was verified. Lastly, we also evaluated ALDH1A3 expression in HCT116 and for U87MG upon NR6 treatment. After 4 h-treatment, NR6 (1 μM and 10 μM) reduces significantly the expression of ALDH1A3 in both cell lines compared to the controls, using qPCR (Figure 7I,J) and western blot (Figure 7K,L) highlighting the effect of the molecule on the enzyme.

## 3. Discussion

Despite the ongoing improvements in the development of new drugs, several tumors still lack a targeted therapy. The ALDH1A3 is a well-known characterizing gene of glioblastoma, and its role and its over expression is correlated with alterations in the glycolysis/gluconeogenesis pathway. ALDH1A3 has been highlighted as a promising target for cancer treatment [23,27,28,29] and is recognized as a CSC marker. CSCs abundance are correlated with chemoresistance to the classical chemotherapies and with invasiveness and, consequently, with high malignancy. We believe that the development of a selective inhibitor of ALDH1A3, could lead to the investigation of a possible co-administration with the Temozolomide [36], the only specific drug for glioblastoma treatment, to increase the final effect of the already existing molecule, reducing the side effects. In this respect, the progenitor of the imidazo[1,2-α]pyridine series, GA11, may be already a promising candidate given its good inhibitory potency. However, the compound is devoid of any selectivity among the different ALDH1A isoforms. Through a rational ligand optimization described elsewhere [27,29], we obtained a potent and selective inhibitor, namely NR6, able to inhibit selectively the ALDH1A3 isoform and to induce cell death, blocking invasiveness and reducing expression of CSC markers. Compared to the parent compounds, NR6 conserves the same heterocycle scaffold with the addition of a cyanide group in the meta position of the 6-phenyl ring. Thanks to the presence of this additional substituent, NR6 binds the protein with a unique arrangement, stabilized by the interaction with several hydrophobic residues. In particular, the interaction with the Y472 residue produces a strong edge-to-face π-π stacking that stabilizes the molecule at the entrance of the catalytic pocket. Even if the site appears partially occupied by the inhibitor, it prevents the binding of the cyclohexene ring belonging to the enzyme natural substrate. In addition, the hydroxyl group of Y472 stabilizes this specific binding mode thanks to a hydrogen bond with the N1 of NR6, that also interacts with a water molecule coordinated with the G136. Interestingly, our sequence analysis demonstrates that Y472 is present in ALDH1A3 and is non-conserved in all the other ALDHs isoforms (Figure S11). This is surprising, in particular for what concerns ALDH1A1 and ALDH1A2 that physiologically metabolize the same substrate, with similar K_M_ [6,37]. We believe that this residue is responsible for the high specificity of NR6 versus ALDH1A3, and the computational analysis strongly confirmed our hypothesis. The effect of NR6 on cancer cells has been analyzed in vitro using ALDH1A3-expressing human glioblastoma and colorectal cancer cells. ALDH1A3 activity on human glioblastoma U87MG and colorectal HCT116 cancer cell lines was investigated by transiently transfecting them with shALDH1A3 and comparing the results with negative controls (HEK293T positive to ALDH1A2, hASTRO and CCD-8Co positive to ALDH1A1 and 4T1 negative to ALDH1A subfamily). NR6 was tested through a viability assay and we determined the high picomolar range in which the inhibitor is able to induce cell death (EC_50_: 378.61 pM for U87MG and 648.26 pM for HCT116 at 72 h). The results have been emphasized by the use of the ALDEFLUOR assay in which ALDH activity is diminished following NR6 treatment, however to a lower extent compared to control cells treated with the pan-ALDH inhibitor DEAB. We also evaluated the effect of NR6 on the metastatic potential in vitro. Performing wound healing and cell invasion assays, using a non-cytotoxic concentration of NR6 at 24 h, we observed a significant reduction of cell migration and invasion, providing the evidence that ALDH1A3 may be one of the mechanisms by which tumor cells migrate from the primary tumor and systemically invade other sites. Notably, ALDH1A3 is not overexpressed in the paraneoplastic tissues of different tumors [23]. To corroborate the idea of ALDH1A3 involvement on CSCs functionality, CSC marker were evaluated. We observed a significant down-expression of *Nestin*, *Nanog1* and *Prom1*. Moreover, the down-expression of *Cd44*, especially on U87MG cell line confirmed the ability of ALDH1A3 inhibitor to interfere with mesenchymal CSCs in glioblastoma. Also, NR6 can be investigated, through target validation mass spectrometry techniques, as a potential AKR inhibitor due to the conserved mechanism, substrates specificity with the ALDHs and a well-known altered expression in several tumors. Overall, we described the structure of the ALDH1A3-NR6 complex, highlighting a peculiar residue able to guarantee a marked selectivity toward the ALDH1A subfamily. Moreover, we were able to describe the ability to induce cytotoxic effect and reduce cell motility and stemness only in our positive control, without any significant effect on the ALDH1A3 negative cell lines. Hence, NR6 represents a starting point for the design and synthesis of more potent ALDH1A3 inhibitor that could be used in combination therapy with other drugs targeting glioblastoma and colorectal cancers.

## 4. Materials and Methods

### 4.1. Crystallization and Structure Determination

Crystals of ALDH1A3 in complex with NR6 was obtained by using the vapor-diffusion technique in sitting drop and applying a spare-matrix-based strategy with a crystallization robot (Oryx4, Douglas Instruments, Hungerford, UK). The best crystals of NR6-ALDH1A3 were grown by mixing 0.5 μL of protein solution at a concentration of 8 mg/mL, preincubated with 1 mM NAD^+^ and 300 μM inhibitor, with an equal volume of a reservoir solution containing 2.4 M sodium malonate, pH 7.0, and equilibrated against 50 μL of the reservoir solution, at 20 °C in about 30 days. For X-ray data collection, crystals were quickly equilibrated in a solution containing the crystallization buffer and 12.5% glycerol as cryo-protectant and flash frozen at 100 K in liquid nitrogen. Data up to 2.95 Å resolution were collected at the beamline ID30A European Synchrotron Radiation Facility (ESRF, Grenoble, France). Analysis of the diffraction data set allowed us to assign the crystal to the orthorhombic P2_1_22_1_ space group with cell dimensions of a = 83.162 Å, b = 89.509 Å, c = 158.646 Å and α = β = γ = 90°, containing two molecules per asymmetric unit with a corresponding solvent content of 53.28%. Data were processed using the program package XDS [44] and the CCP4 suite of programs [45] was used for scaling. Determination of the structure of NR6-ALDH1A3 was carried out by means of the molecular replacement technique using the coordinates of the tetramer of human ALDH1A3 as the search model (PDB code: 5FHZ. [40]). PHASER [46] was used to automatically determine the ALDH1A3 structure in complex with the compound. The initial model was subjected to iterative cycles of crystallographic refinement with the programs REFMAC5 (CCP4 Program Suite v7.1.010) [47] and PHENIX.REFINE v. 1.19-4092 [48], alternated with manual graphic session for model building using the program Coot [49]. Then 5% of randomly chosen reflections were excluded from refinement of the structure and used for the Free R factor calculation [50]. The program ARP/wARP (CCP4 Program Suite v7.1.010) [51] was used for adding solvent molecules. Refinement was continued until convergence to R-factor and free R-factor values of 0.1930 and 0.2510 for NR6-ALDH1A3. Data collection and refinement statistics are given in Table S1. The atomic coordinates and structure factors of human ALDH1A3 in complex with NR6 have been deposited with the Protein Data Bank (www.rcsb.org) with the accession codes 7A6Q.

### 4.2. Molecular Dynamic Simulation

The crystallographic structure of the ALDH1A3/NR6 complex solved in this work was used as starting point in MD simulations. The protein was parametrized by the ff14SB force field [52] while the NR6 molecule was parametrize by the general amber force field 2 (GAFF2). The complex was then solvated in a rectilinear box of TIP3P water molecules and the solvent was energy minimized for 5000 steps of which the firs 1500 using the Steepest Descent algorithm (SD) and the last 3500 using the Conjugate Gradient algorithm (CG), while keeping the complex frozen. Then, 10,000 steps of energy minimization were carried out on the whole system applying the SD for the first 1500 steps, and the GC for the remaining 8500 steps. The system was then heated from 0 to 300 K over 1 ns at constant volume by the Langevin thermostat (NVT ensemble), and the system density was equilibrated over 1 ns at constant pressure by the Berendsen barostat (NPT ensemble). After a 50 ns of preliminary equilibration, 500 ns of unrestrained MD trajectory was produced by the AMBER version 18 program [53], and processed by the CPPTRAJ software version 18.00 [54] In order to estimate the theoretical affinity of binding of the complex, and the energy contribution of Y472, 100 representative frames were extracted from MD trajectory by cluster analysis and were submitted to MM-GBSA alanine scanning.

### 4.3. Cell Culture

U87MG human glioblastoma, CCD-18Co human healthy colon fibroblasts and 4T1 murine mammary carcinoma cell lines were cultured in Minimum Essential Medium Eagle (MEM, Sigma-Aldrich). HEK293T human embryonic kidney cell line was cultured in Dulbecco’s Modified Eagle’s Medium (DMEM, Sigma-Aldrich, St. Louis, MO, USA). HCT116 human colorectal cancer was cultured in McCoy’s 5a Medium Modified (Sigma-Aldrich). All these cell lines were purchased from ATCC. Media were supplemented with 10% fetal bovine serum (FBS, Gibco, Waltham, MA, USA), 2 mg/mL glutamine, 10 U/mL penicillin and 100 g/mL streptomycin (Sigma-Aldrich). Human foetal astrocytes were kindly provided by Eleonora Aronica’s lab in Amsterdam and cultured in DMEM+F10 medium. Cells were maintained in a controlled atmosphere of 5% CO2 with humidity at 37 °C. Cell were detached from plates by trypsin-EDTA (Sigma-Aldrich). 3D spheroids were cultured in 1% agarose-coated 96-Multiwell. After 48 h formation, 5 × 10^4^ cell spheroids were treated with NR6 at decreasing concentration and cell viability evaluated with MTT.

### 4.4. Creation of U87MG and HCT116 shALDH1A3 Cell Line

U87MG human glioblastoma and HCT116 human colorectal cancer were transiently transfected with short harpin RNA (shRNA). Two different plasmid system were used: GIPZ Lentiviral shRNA clone V3LHS_378585 (#85) and clone V3LHS_378584 (#84) (GEDharmacon, Lafayette, CO, USA). The transient transfection was conducted in presence of Lipofectamine 2000 (Invitrogen, Carlsbad, California) and OptiMEM (Sigma-Aldrich), according to manufacturer’s instruction, in MW24 with 10 × 10^5^ U87-MG/well. The growth of transfected cells was monitored at time 0, 24 h, 48 h and 72 h.

### 4.5. Cell Viability Assay

10 × 10^5^ U87MG human glioblastoma, HCT116 human colorectal cancer, HEK293T human embryonic kidney, CCD-18Co human colon fibroblasts, 4T1 murine mammary carcinoma and human foetal astrocytes cell lines were plated in respective medium and treated for 72 h with the indicated compounds, solubilized in DMSO (in a final concentration of 10%). The colorimetric 3-(4,5-dimethylthiazol-2-yl)-2,5-dipehenyltetrazolium bromide (MTT) method to analyze cell viability was used. Briefly, cells were plated in 24-well plates and treated as indicated for the appropriate time. Cells were washed once in Locke buffer and 300 µl of MTT (250 µg/mL in Locke buffer) were added before returning the cells to the incubator for 1 h to allow the formation of the purple formazan crystals. After 1 h, 600 µl of isopropanol/0.1 M HCl were added to each well, and the absorbance was read at 570 nm in a plate reader (Victor3V, PerkinElmer Life Sciences, Waltham, MA, USA).

### 4.6. Aldefluor Assay

The Aldefluor kit (Stem Cell Technologies, Vancouver, BC, Canada) was used to analyse and identify cell populations with high ALDH enzymatic activity. Briefly, 1 × 10^6^ cells/mL were suspended in Aldefluor assay buffer containing the ALDH substrate BODIPY-aminoacetaldehyde and incubated at 37 °C for 45 min. For each sample, cell aliquots were incubated with or without 50 µM diethylaminobenzaldehyde (DEAB), an ALDH-specific inhibitor and 1 μM NR6. Then, the cells were analysed on (S3e Cell Sorter, BioRad, Hercules, CA, USA) and with FlowJo program v10.7.

### 4.7. Statistical Analysis

Data are presented as mean ± SEM. The normality of data distributions was assessed using Shapiro–Wilk test. Parametric (unpaired *t*-test and One-way analysis of variance (ANOVA) followed by Tukey’s post-hoc) or non-parametric (Mann-Whitney U test and One-way Kruskal-Wallis H test followed by Dunn’s post-hoc) statistical analysis were used for comparisons of data. All statistical assessments were two-sided and a value of *p* < 0.05 was considered statistically significant. Statistical analysis was performed using GraphPad Prism software (GraphPad Software, v8.4.3). The *n* number of independent experiments (defined as experiments performed on different days) is reported in the respective figure legends. The Graphical Abstract was created with BioRender.com (https://biorender.com/).

### 4.8. Additional Materials and Methods

Additional Materials and Methods are reported in Supplementary Information, including uncropped Western Blot Images (Figure S12).

## 5. Conclusions

In the present manuscript, we describe a series of multidisciplinary experiments that allow us to demonstrate NR6 as the first ALDH1A3 potent and selective inhibitor. Selectively targeting CSCs through the specific ALDH1A3 inhibition may be a therapeutic winning strategy to hinder invasiveness and stemness of cancer cells and consecutively tumour growth. For the first time, we highlight the residue for being selective on ALDH1A3, that, surely, will improved the development of inhibitors on this isoform, and might be combined with the classical therapies already in use.

## Data Availability

The atomic coordinates and structure factors of human ALDH1A3 in complex with NR6 have been deposited with the Protein Data Bank (www.rcsb.org) with the accession codes 7A6Q.

## Supplementary Materials

The following are available online at https://www.mdpi.com/2072-6694/13/2/356/s1, Figure S1. (A) Representative Western Blot (right) and quantification (left) of shALDH1A3 knockdown of U87MG cells. *n* = 2 independent experiments. (B) qPCR of Aldh1a3 in U87MG cells, *n* = 2 independent experiments (C) Cellular growth of shALDH1A3 U87MG cells (using two shRNA, #84 and #85) compared to SCR. 4 replicates *n* = 4 independent experiments. (D) Representative Western Blot (right) and quantification (left) of shALDH1A3 knockdown of HCT116 cells. *n* = 2 independent experiments. (E) qPCR of Aldh1a3 in U87MG cells, *n* = 2 independent experiments (F) Cellular growth of shALDH1A3 HCT116 cells (using two shRNA, #84 and #85) compared to SCR. 4 replicates of *n* = 4 independent experiments. (*** *p* < 0.001, ** *p* < 0.01 by unpaired two-tailed *t*-test). Figure S2. (A) histograms from the high-throughput screening of the three imidazo[1,2-*a*]pyridine. (B) Chemical structure of the test compounds and values of the residual activity of ALDH1A1, 1A2 and 1A3 in complex with the three inhibitors. (C) IC_50_ and K_i_ values of three complexes. Figure S3. Michaelis-Menten and Lineweaver-Burk graphs of ALDH1A3-NR6 complex. The graphs clearly define a competitive mechanism of inhibition. Figure S4. (A) Cartoon representation of the superimposition between the ALDH1A3 in complex with NR6 (PDB code 7A6Q), ALDH1A1 (PDB code 4WB9) [7] and ALDH1A2 (6B5G) [8]. All the three domains are highlighted (catalytic, NAD^+^ and oligomerization). Figure S5. (A) Protein expression of cleaved caspase-3 and inactive caspase-3 of U87MG and HCT116 treated with NR6 at 10 and 1 µM, for 24 h. (B) The intensity of each band was quantified by densitometry, and the data were normalized to actin. Figure S6. (A) Gating strategy used to analyse ALDEFLUOR assay. Figure S7. ALDEFLUOR assay in (A) HEK293T, (B) hASTRO and (C) 4T1 cells respectively, treated with ALDEFLUOR probe only (on the left), DEAB as positive control (in the center) and with 10 μM NR6 (on the right). Figure S8. (A) Dose-response curves of U87MG and HCT116 cell lines treated with NR6 for 24 h. (B) EC_50_ table values. EC_50_s were calculated with concentration–response curves and using Kaleidograph software. Data shown are mean percentages ± SD of three independent experiments (*n* = 12 for viability). Figure S9. (A) Representative wound healing images of 4T1 cells treated with vehicle and NR6 10 nM. (B) Percentage of 4T1 wound closure (compared to % of the control) after 24 h of treatment. Figure S10. (A) Representative wound healing images of U87MG cells SCR and transfected with shALDH1A3. (B) Representative wound healing images of HCT116 cells SCR and transfected with shALDH1A3. (C) Percentage of U87MG wound closure (compared to % of SCR) after 24 h of treatment. (D) Percentage of HCT116 wound closure (compared to % of SCR) after 24 h of treatment. (E) Gene expression analysis quantified by qPCR of *Nes*, *Nanog1*, *Cd44* and *Prom1* in U87MG and (F) in HCT116. Figure S11. (A) Sequence alignment between all the nineteen isoforms. The tyrosine is not conserved in any other ALDH isoenzymes. None of the residues close to the same region present a hydrophobic side chain that could supply the absence of the tyrosine to coordinate the heterocycle with the same kind of interaction (edge-to-face π-π stacking). Figure S12. Uncropped western blot figures. Table S1. Data collection and refinement statistics of the three-dimensional structure of human ALDH1A3 in complex with NR6.
