# Supplementary material for: A Selective Competitive Inhibitor of Aldehyde Dehydrogenase 1A3 Hinders Cancer Cell Growth, Invasiveness and Stemness In Vitro
Source: Cancers (Basel). 2021 Jan 19;13(2):356. doi: 10.3390/cancers13020356 (PMC7835878; doi:10.3390/cancers13020356)
Supplement: Supplementary file 1 [file cancers-13-00356-s001.pdf]

cancers

MDPI

Supplementary Materials

A Selective Competitive Inhibitor of Aldehyde Dehydrogenase 1A3 Hinders Cancer Cell Growth, Invasiveness and Stemness In Vitro

Edoardo L. M. Gelardi, Giorgia Colombo, Francesca Picarazzi, Davide M. Ferraris, Andrea Mangione, Giovanni Petrarolo, Eleonora Aronica, Menico Rizzi, Mattia Mori, Concettina La Motta, Silvia Garavaglia*

Supplementary materials

1. Extended Materials and methods

1.1. Chemistry

The imidazo[1,2-*a*]pyridine derivatives NR2, NR4 and NR6, were synthesized at the Department of Pharmacy of the University of Pisa, Italy, following a previously reported procedure [1,2]. Briefly, a mixture of 5-bromopyridin-2-amine (1.00 mmol), 2-bromo-1-phenylethan-1-one (1.00 mmol) and potassium carbonate (1.00 mmol) was refluxed under stirring in ethanol until the obtainment of the key intermediate 6-bromo-2-phenylimidazo[1,2-*a*]pyridine, which provided the target inhibitor NR2, NR4 and NR6 by a coupling reaction with the suitably substituted phenyl boronic acid, in the presence of $Pd(OAc)_2$and PPh_3 as the catalysts.

2-(2-Phenylimidazo[1,2-*a*]pyridin-6-yl)benzonitrile, NR2. White solid. Mp 213–215 °C. Cryst. solvent: EtOH. Yield: 26%. ^{1}H-NMR (δ, ppm): 8.857 (s, 1H), 8.855 (s, 1H), 8.852 (d, 3H, J = 7.76 Hz), 7.879 (t, 1H, J = 7.64 Hz), 7.775–7.740 (m, 2H), 7.655 (t, 1H, J = 7.68 Hz), 7.475 (t, 3H, J = 7.72 Hz), 7.355 (t, 1H, J = 7.37Hz).

4-(2-Phenylimidazo[1,2-*a*]pyridin-6-yl)benzonitrile, NR4. White solid. Mp 209–210 °C. Cryst. solvent: Acetonitrile. Yield: 39%. ^{1}H-NMR (δ, ppm): 9.066 (s, 1H), 8.436 (s, 1H), 8.017 (d, 2H, J = 7.08 Hz), 7.979 (d, 4H, J = 10.20 Hz), 7.729 (d, 1H, J = 9.37 Hz), 7.690 (dd, 1H, J = 10.16 Hz, J = 1.60 Hz), 7.472 (t, 2H, J = 7.60 Hz), 7.354 (t, 1H, J = 7.40 Hz).

3-(2-Phenylimidazo[1,2-*a*]pyridin-6-yl)benzonitrile, NR6. White solid. Mp 170–171 °C. Cryst. solvent: EtOH. Yield: 30%. ^{1}H-NMR (δ, ppm): 9.043 (s, 1H), 8.415 (s, 1H), 8.272 (s, 1H), 8.110 (d, 1H, J = 8.00 Hz), 8.022 (d, 2H, J = 7.30 Hz), 7.881 (d, 1H, J = 7.80 Hz), 7.737 (d, 2H, J = 7.77 Hz), 7.707 (s, 1H), 7.470 (t, 2H,J = 7.52 Hz), 7.352 (t, 1H, J = 7.28 Hz).

1.2. Expression and Purification of Human ALDHs

A common protocol to express and purify all three members of the ALDH1A subfamily has been optimized [2,3]. Briefly, induced cells, collected by centrifugation, were re-suspended in an appropriate volume of lysis buffer (50 mM Na_2HPO_4, 300 mM NaCl, 1 mM β-mercaptoethanol, 20 mM imidazole, pH 7.5) added of benzonase nuclease (250 U/ µL) and Protease inhibitor cocktail from SIGMA. After ultracentrifugation, the clarified sample was purified by a His-tag affinity chromatography followed by size-exclusion chromatography, using an AKTA FPLC system, at 4°C. To evaluate the purity and homogeneity of the protein, after each purification step, eluted fractions were analyzed by SDS-PAGE and the protein quantification was always determined by Bradford protein assay. This procedure allowed us to obtain about 20 mg of pure and active human ALDH1A3, ALDH1A2 and ALDH1A1 used for all crystallization trials and kinetic analyses.

1.3. High-throughput screening to determine Inhibitory Efficacy of Imidazo[1,2-a]pyridine Derivatives

All the three imidazo[1,2-*a*]pyridine derivatives were screened as inhibitors of ALDH1A3, ALDH1A1, and ALDH1A2 isoenzymes, using the previously reported continuous spectrometric assay for ALDH1A3 optimized to suit Greiner Bio-One 96-UV-Transparent Microplate [4]. Tests were carried out by using 100 μL of reaction mixture, containing 20 mM Tris HCl pH 8.0, 1 mM β-mercaptoethanol, 150 mM KCl, 15% DMSO 500 μM NAD^+, and 2.6 μM pure recombinant isoenzymes. Reactions were started by the addition of 20 mM acetaldehyde. Changes in absorbance at 340 nm (εNADH = 6220 M–1 cm–1) were monitored for 30 min in Tecan Sunrise spectrophotometer, at 25 °C. The activity of the three enzymes was tested in the presence of each synthesized compound, at 25 μM concentration in triplicates. To avoid any possible error in the data analysis, in every plate was tested the natural NAD^+ reduction, using the same mix without the addiction of acetaldehyde. The intrinsic signal was subtracted from every data analysis.

1.4. Enzyme Kinetic Analysis to Calculate IC_{50} and K_i Values of the compounds

Selected compounds showing the best inhibitory activity at 25 μM were further investigated for their inhibitory efficacy, to calculate their IC_{50} values. The enzymatic inhibition assays were performed in a total volume of 200 μL of 20 mM Tris HCl pH 8.0, 1 mM β-mercaptoethanol, 150 mM KCl, 15% DMSO, 500 μM NAD^+, 2.6 μM of pure recombinant isoenzymes in the presence of different inhibitors concentrations from 100 μM to 0.78125 μM and preincubated for 5 min. To calculate the K_i value of NR6 in complex with all the three ALDH1A isoenzymes, the activity was tested in presence of different concentrations of the substrate and inhibitor, to understand the mechanism of inhibition and calculate the K_i value. The enzymatic inhibition assays were performed in a total volume of 100 μL of 20 mM Tris HCl pH 8.0, 1 mM β-mercaptoethanol, 150 mM KCl, 15% DMSO, 500 μM NAD^+, 2.6 μM of pure recombinant isoenzymes in the presence of different inhibitors concentrations from 200 μM to 1 μM and different concentrations of acetaldehyde from 20 mM to 1 mM for ALDH1A2 and ALDH1A3 and from 1 mM to 0.2 mM for ALDH1A1. The kinetic parameters were determined by fitting the measured data to a Michaelis–Menten curve [5] by using SigmaPlot (SigmaPlot Extract Graphs and Data Analysis (StatSys v12.3)).

1.5. Western Blotting Analysis

Cells were lysed in lysis buffer composed of 20 mM HEPES, 100 mM NaCl, 5 mM EDTA, 1% Nonidet P-40+ Protease & Phosphatase Inhibitor Cocktail (Sigma). Proteins quantification was performed with Bradford Reagent (Sigma-Aldrich), and proteins were resolved on SDS–PAGE and transferred with TurboBlot system (BioRad). Densitometry analysis was performed with ImageLab program (Bio-Rad).

1.6. Reagents

Antibodies: Mo anti-actin A1978 Sigma, Rb anti-ALDH1A3 ab129815 AbCam.

1.7. Gene Expression Analysis

Cells were lysated with Trizol reagent (Life-Technologies) and extracted with chloroform. 1μg mRNA was reverse transcribed with SENSIFAST kit as manufacturer's protocol (Aurogene) and cDNA expression determined with qPCR using SYBR-green (Bio-Rad) and detected by the CFX96 Real-Time System (Biorad). Expression data were normalized to actin expression.

The sequences of gene-specific primers are listed in the Table below.

Primer	Forward 5′ -> 3′	Reverse 5′ -> 3′
h*Aldh1a1*	GGCATGATTCAGTGAGTGGC	TCCTTCTTCTACCTGGCAGAG
h*Aldh1a2*	AGACTGTTTGGTTAAAATCCCCA	GGAACACTCTCCCACTCTCT
h*Aldh1a3*	TGGATGCCCTGAGTCGTG	GGCTTCCCTGTATCCATCGT
m*Aldh1a1*	TGACAAATCACCGCACTGC	TTTGGTCCTGGCTCTGTGG

m*Aldh1a2*	CACGCCCAACCTCGAGAT	GGAACACTCTCCCACTCTCT
m*Aldh1a3*	TTGTAGAAAGGGACCGAGCG	TCCTGCCCTGGATTTTGTCT
h*Nes*	CAGGAGAAACAGGGCCTACA	GCTGAGGGACATCTTGAGGT
h*Nanog1*	TCTTCCACCAGTCCCAAAGG	TTTCTTGACCGGGACCTTGT
h*Cd44*	CGCTTTGCAGGTGTATTCCA	GCAGGTCTCAAATCCGATGC
h*Prom1*	CCACAGATGCTCCTAAGGCT	CTTCTGGGAAATCACGCGG

1.8. Wound Healing Assay

When confluent monolayers of U87, HCT116 and 4T1 cells were established, we performed a cross-shaped scratch with tip, as reported in [6]. Then, the cells were washed twice with PBS to remove residual cell debris. Cells were then incubated with NR6 (10 nM; concentrations that occurs less mortality in 24h) for 24 h and pictures of a defined wound spot were made at different time points. The area of the wound in the microscopic pictures was measured and analysed using Image J software (National Institutes of Health, MD, USA).

1.9. Cell Invasion Assay

3x10[4] cells (U87 and HCT116) were transferred to the top of the Matrigel-coated invasion chambers (BD Biosciences, San Jose, CA, USA) in a serum-free medium. Respective media containing 10% fetal bovine serum were added to the lower chamber, in presence or not of NR6 (10 nM). After 24 h, the non-invading cells were removed, and the invading cells were fixed using methanol, stained with 0.1% crystal violet and images were captured under a 40x magnification. The tests were repeated in three independent experiments.

Supplementary Figures:

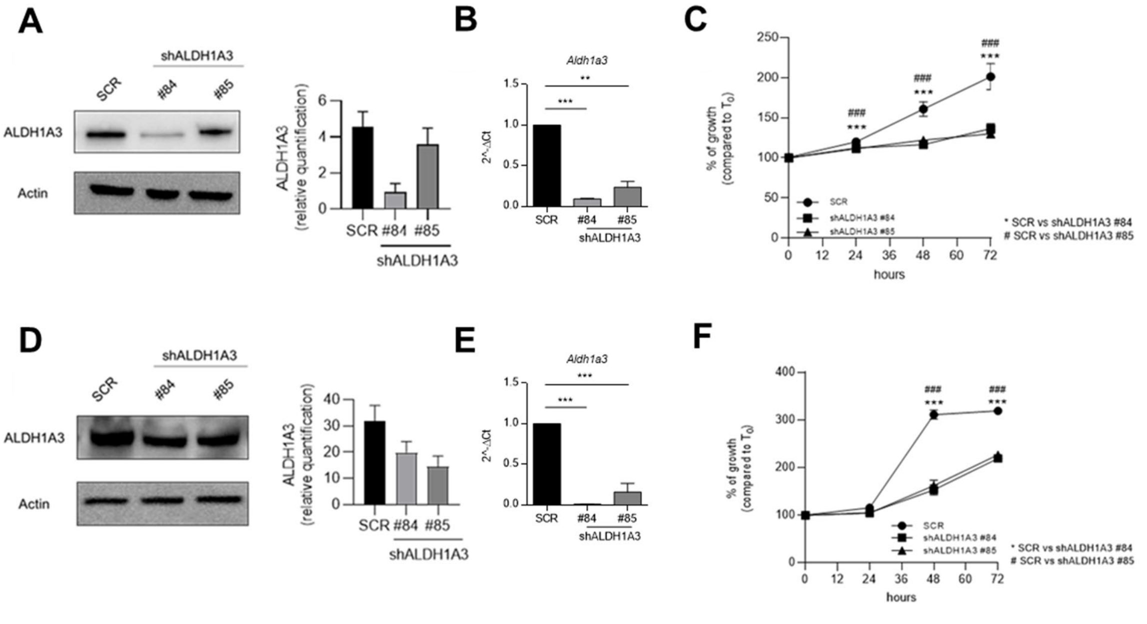

Figure S1. (**A**) Representative Western Blot (right) and quantification (left) of shALDH1A3 knockdown of U87MG cells. *n* = 2 independent experiments. (**B**) qPCR of Aldh1a3 in U87MG cells, *n* = 2 independent experiments (**C**) Cellular growth of shALDH1A3 U87MG cells (using two shRNA, #84 and #85) compared to SCR. 4 replicates *n* = 4 independent experiments. (**D**) Representative Western Blot (right) and quantification (left) of shALDH1A3 knockdown of HCT116 cells. *n* = 2 independent experiments. (**E**) qPCR of Aldh1a3 in U87MG cells, *n* = 2 independent experiments (**F**) Cellular growth

of shALDH1A3 HCT116 cells (using two shRNA, #84 and #85) compared to SCR. 4 replicates of $n = 4$ independent experiments. (*** $p < 0.001$, ** $p < 0.01$ by unpaired two-tailed T-test). .

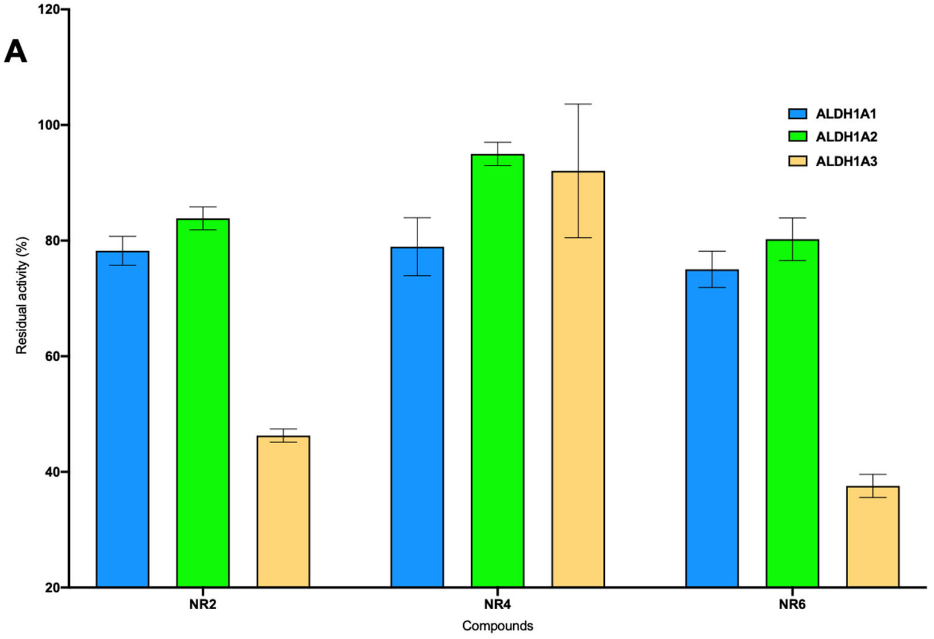

B

Residual activity at 25 μM (%)

NR2	NR4	NR6
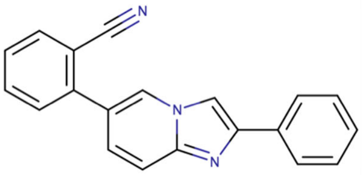	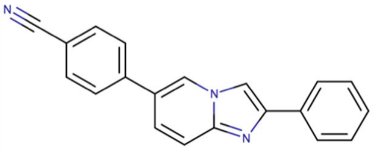	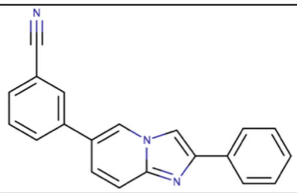
ALDH1A1= 78.2 ± 2.5 %<br>ALDH1A2= 83.7 ± 2.0 %<br>ALDH1A3= 46.3 ± 1.1 %	ALDH1A1= 79.0 ± 5.0 %<br>ALDH1A2= 95.0 ± 2.0 %<br>ALDH1A3= 92.1 ± 11.6 %	ALDH1A1= 75.0 ± 3.1 %<br>ALDH1A2= 80.2 ± 3.7 %<br>ALDH1A3= 37.6 ± 2.0 %

Biochemical characterisation

C

	ALDH1A1	ALDH1A2	ALDH1A3
IC_{50} (μM)	57.6 ± 2.2 (R^2= 0.95)	123.6 ± 2.2 (R^2= 0.96)	5.3 ± 1.5 (R^2= 0.97)
K_i (μM)*	262.2 ± 76.4 (R^2= 0.96)	257.6 ± 26.4 (R^2= 0.98)	3.7 ± 0.4 (R^2= 0.97)

*full competitive inhibitor

Figure S2. (**A**) histograms from the high-throughput screening of the three imidazo[1,2-*a*]pyridine. (**B**) Chemical structure of the test compounds and values of the residual activity of ALDH1A1, 1A2 and 1A3 in complex with the three inhibitors. (**C**) IC_{50} and K_i values of three complexes.

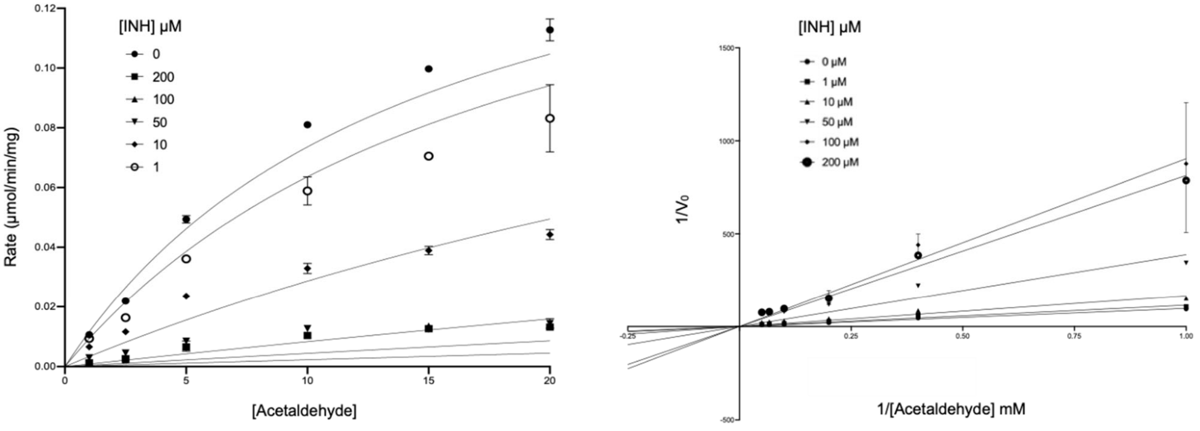

R^2= 0.97

Ki= 3.7 ± 0.4 μM

Figure S3. Michaelis-Menten and Lineweaver-Burk graphs of ALDH1A3-NR6 complex. The graphs clearly define a competitive mechanism of inhibition.

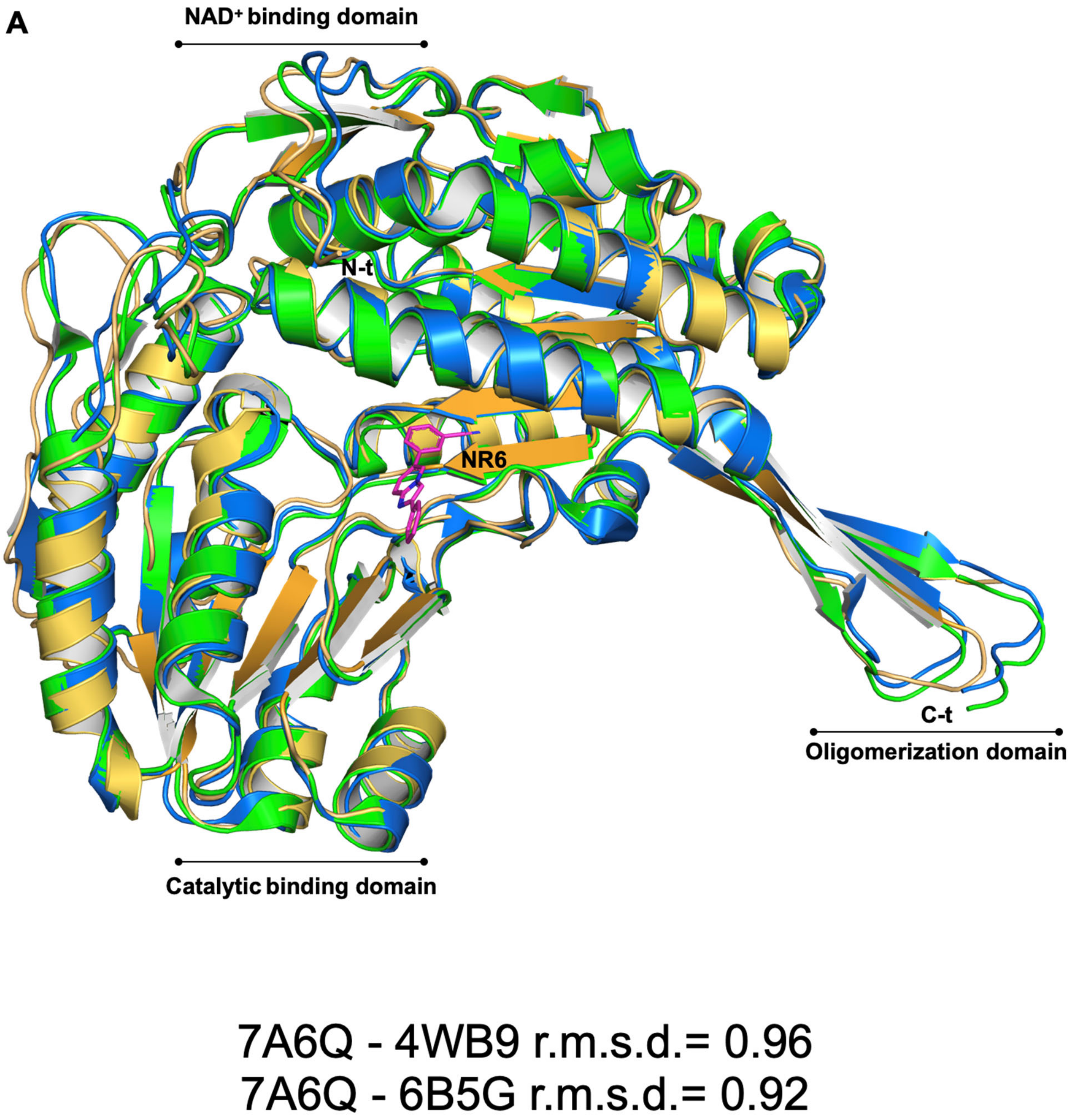

Figure S4. (**A**) Cartoon representation of the superimposition between the ALDH1A3 in complex with NR6 (PDB code 7A6Q), ALDH1A1 (PDB code 4WB9) [7] and ALDH1A2 (6B5G) [8]. All the three domains are highlighted (catalytic, NAD^+ and oligomerization).

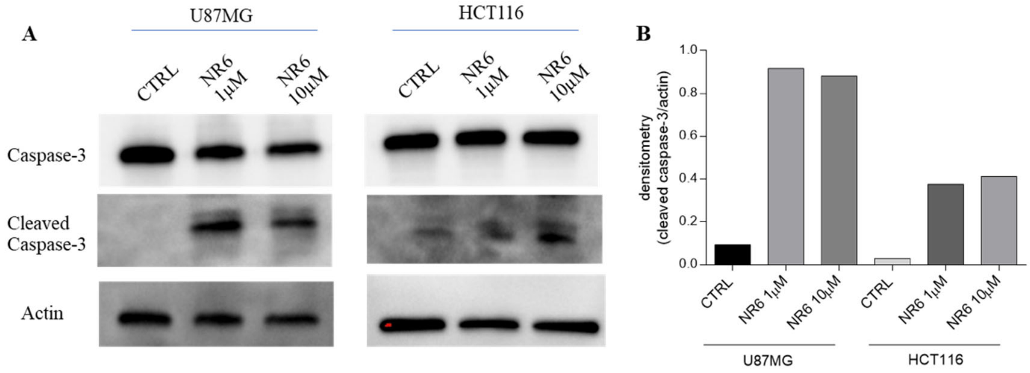

Figure S5. (**A**) Protein expression of cleaved caspase-3 and inactive caspase-3 of U87MG and HCT116 treated with NR6 at 10 and 1μM, for 24h. (**B**) The intensity of each band was quantified by densitometry, and the data were normalized to actin.

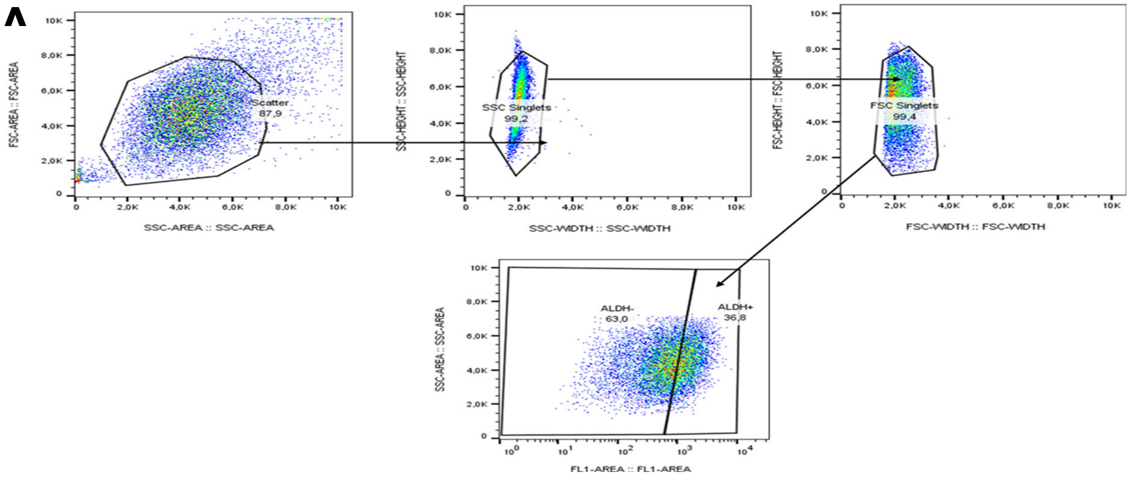

Figure S6. (**A**) Gating strategy used to analyse ALDEFLUOR assay.

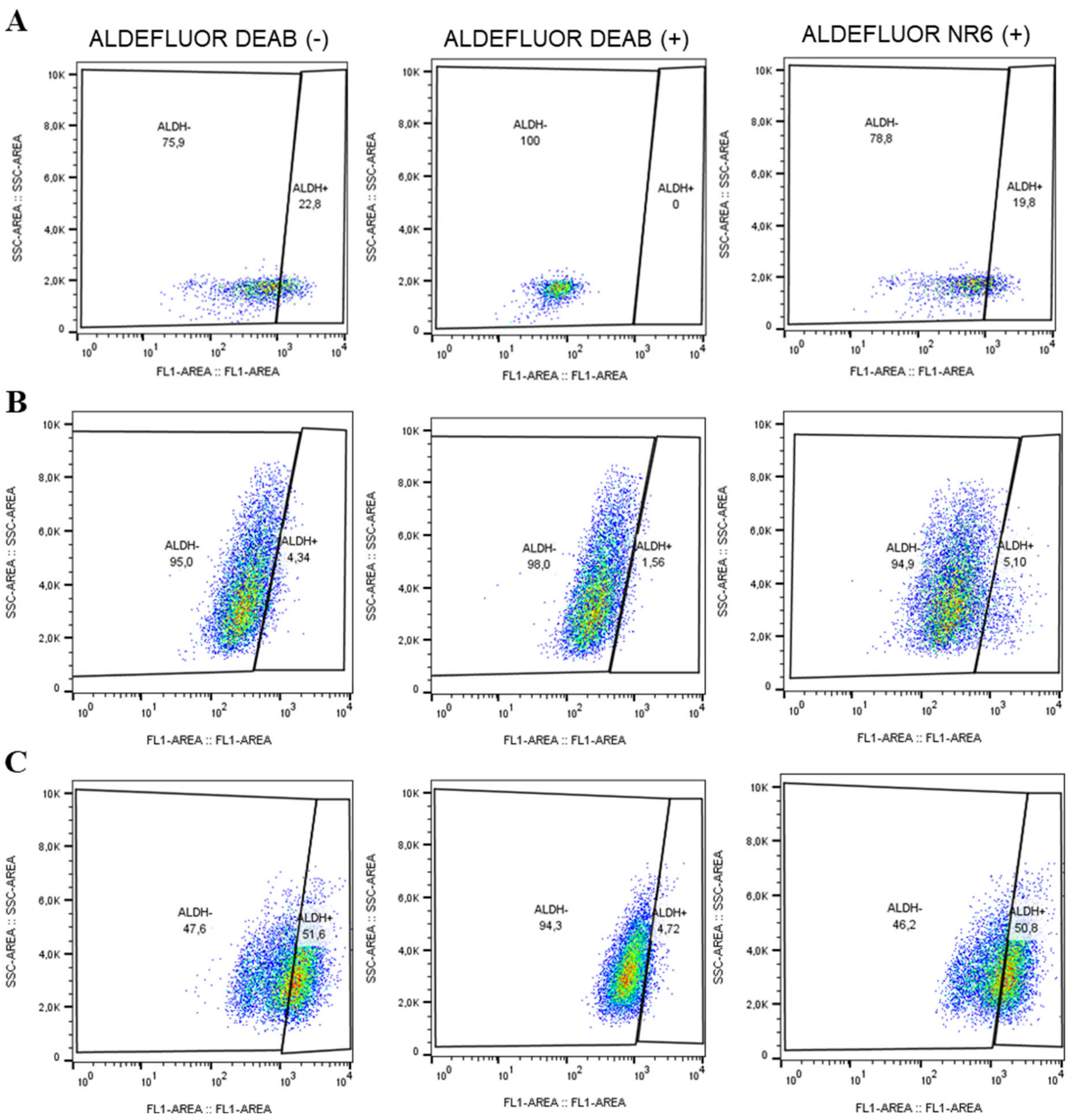

Figure S7. ALDEFLUOR assay in (**A**) HEK293T, (**B**) hASTRO and (**C**) 4T1 cells respectively, treated with ALDEFLUOR probe only (on the left), DEAB as positive control (in the center) and with 10 μM NR6 (on the right).

A

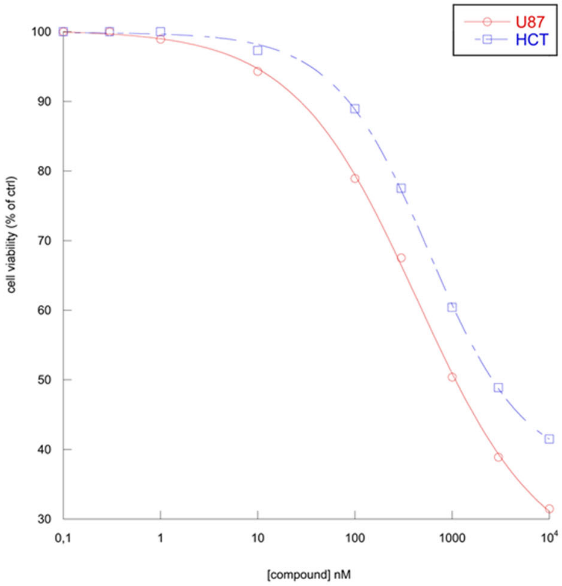

B

Cell line	EC_{50} (nM)
U87	434,7± 4,35
HCT116	576,05± 2,58

Figure S8. (**A**) Dose-response curves of U87MG and HCT116 cell lines treated with NR6 for 24h. (**B**) EC_{50} table values. EC_{50}s were calculated with concentration–response curves and using Kaleidograph software. Data shown are mean percentages ± SD of three independent experiments (n = 12 for viability).

A

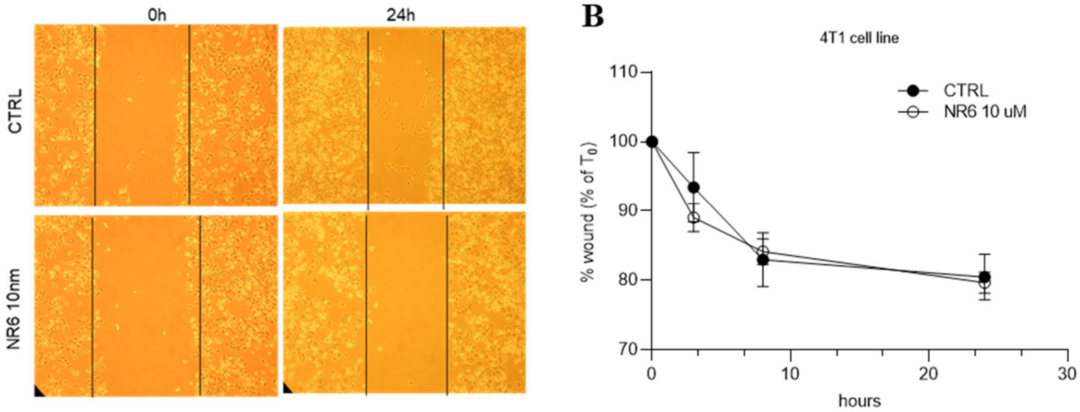

Figure 9. (**A**) Representative wound healing images of 4T1 cells treated with vehicle and NR6 10nM. (**B**) Percentage of 4T1 wound closure (compared to % of the control) after 24 hours of treatment.

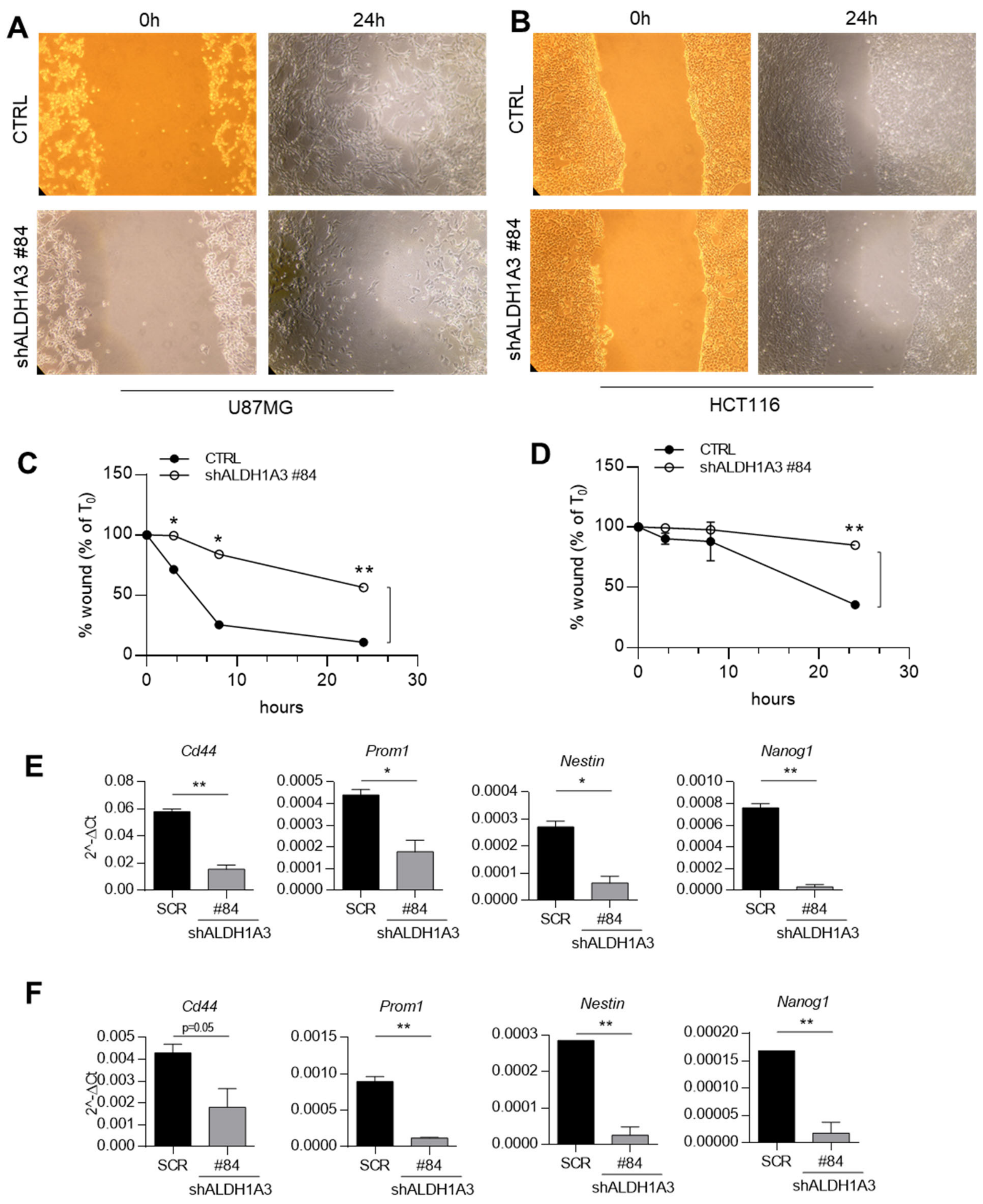

Figure S10. (A) Representative wound healing images of U87MG cells SCR and transfected with shALDH1A3. **(B)** Representative wound healing images of HCT116 cells SCR and transfected with shALDH1A3. **(C)** Percentage of U87MG wound closure (compared to % of SCR) after 24 hours of treatment. **(D)** Percentage of HCT116 wound closure (compared to % of SCR) after 24 hours of treatment. **(E)** Gene expression analysis quantified by qPCR of *Nes*, *Nanog1*, *Cd44* and *Prom1* in U87MG and (**F**) in HCT116.

A

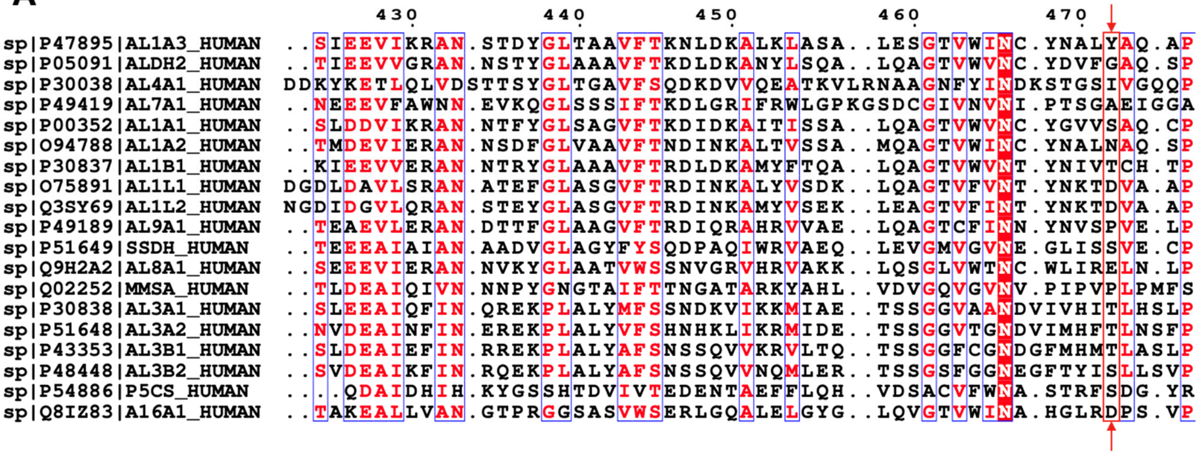

Figure S11. **(A)** Sequence alignment between all the nineteen isoforms. The tyrosine is not conserved in any other ALDH isoenzymes. None of the residues close to the same region present a hydrophobic side chain that could supply the absence of the tyrosine to coordinate the heterocycle with the same kind of interaction (edge-to-face π-π stacking).

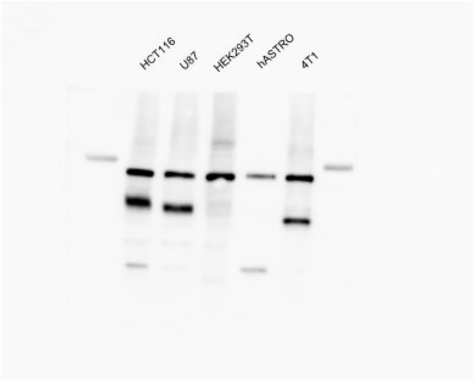

ALDH1A3 blot

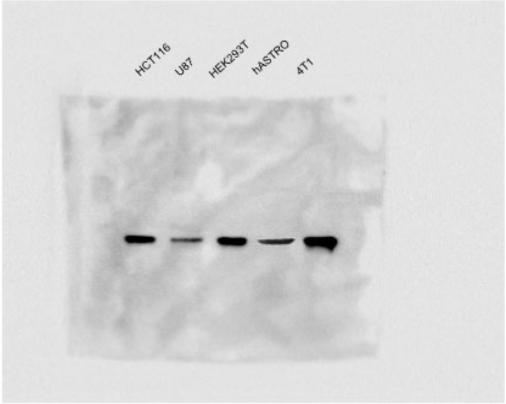

Actin blot

Figure 1B

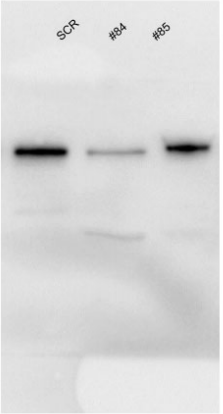

ALDH1A3 blot

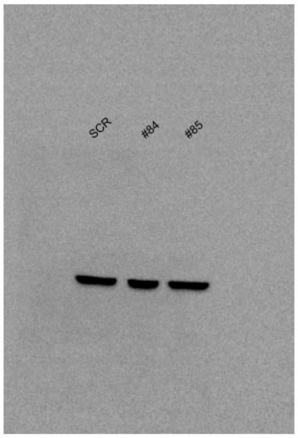

Actin blot

Figure 1C + S1 A

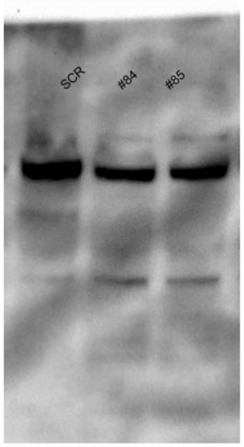

ALDH1A3 blot

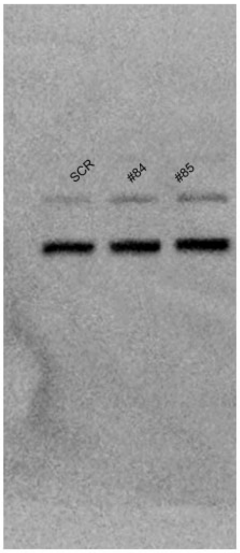

Actin blot

Figure 1E + S1 C

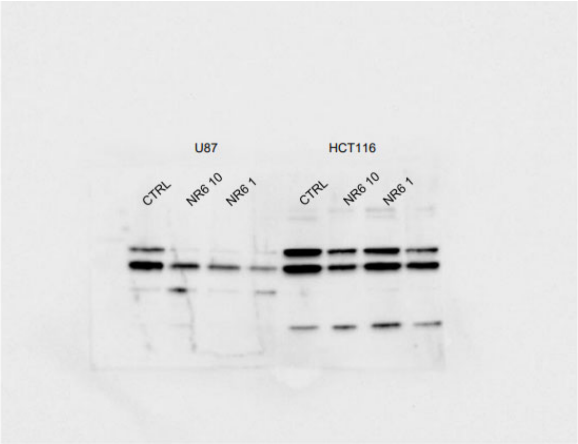

ALDH1A3 blot

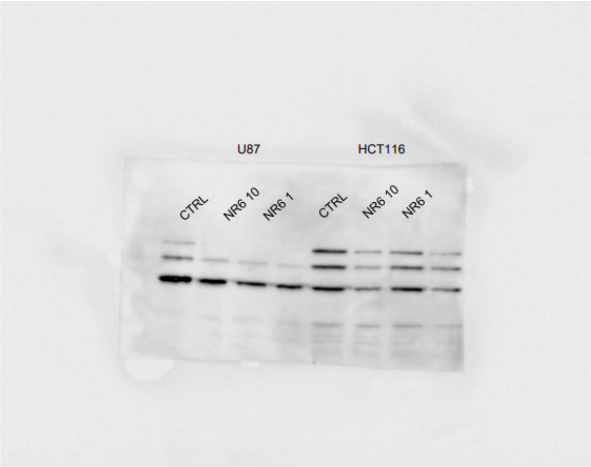

Actin blot

Figure 7K and L (row 4 and 8 no selected)

Figure S12. Uncropped western blot figures.

Supplementary Table:

Table S1. Data collection and refinement statistics of the three-dimensional structure of human ALDH1A3 in complex with NR6.

**Space group**	P 21 2 21
**Cell Dimensions**	
**a, b, c (Å)**	83.162 89.509 158.646
**A, b, c (°)**	90 90 90
**Resolution range (Å)**	48.32 - 2.95
**R pim/ R merge**	0.058 (0.412)/ 0.121 (0.889)
**Mean I/sigma(I)**	10.5 (1.8)
**Completeness (%)**	99.51 (99.56)
**Multiplicity**	4.3 (4.4)
**Refinment**	
**Resolution (Å)**	2.95
**R-work**	0.1930 (0.2833)
**R-free**	0.2510 (0.3553)
**Reflections used in refinement**	25519 (2509)
**Reflections used for R-free**	1305 (118)
**Number of non-hydrogen atoms**	7787
**macromolecules**	7560
**ligands**	189
**solvent**	38
**Average B-factor**	77.46
**macromolecules**	77.71
**ligands**	95.59
**solvent**	57.28
**RMS(bonds)**	5
**RMS(angles)**	0.72
**Ramachandran outliers (%)**	0.10
**Rotamer outliers (%)**	0.00

References:

1. Cheng, P.; Wang, J.; Waghmare, I.; Sartini, S.; Coviello, V.; Zhang, Z.; Kim, S.-H.; Mohyeldin, A.; Pavlyukov, M.S.; Minata, M.; et al. FOXD1-ALDH1A3 Signaling Is a Determinant for the Self-Renewal and Tumorigenicity of Mesenchymal Glioma Stem Cells. *Cancer Res.* **2016**, *76*, 7219–7230, doi:10.1158/0008-5472.CAN-15-2860.
2. Quattrini, L.; Gelardi, E.L.M.; Coviello, V.; Sartini, S.; Ferraris, D.; Mori, M.; Nakano, I.; Garavaglia, S.; La Motta, C. Imidazo[1,2-*a*]Pyridine Derivatives as Aldehyde Dehydrogenase Inhibitors: Novel Chemotypes to Target Glioblastoma Stem Cells. *J. Med. Chem.* **2020**, *63*, 4603-4616, acs.jmedchem.9b01910, doi:10.1021/acs.jmedchem.9b01910.
3. Quattrini, L.; Gelardi, E.L.M.; Petrarolo, G.; Colombo, G.; Ferraris, D.M.; Picarazzi, F.; Rizzi, M.; Garavaglia, S.; La Motta, C. Progresses in the Field of Aldehyde Dehydrogenase Inhibitors: Novel Imidazo[1,2-*a*]Pyridines Against the 1A Family. *ACS Med. Chem. Lett.* **2020**, *11*, 963-970, acsmedchemlett.9b00686, doi:10.1021/acsmedchemlett.9b00686.
4. Moretti, A.; Li, J.; Donini, S.; Sobol, R.W.; Rizzi, M.; Garavaglia, S. Crystal Structure of Human Aldehyde Dehydrogenase 1A3 Complexed with NAD+ and Retinoic Acid. *Sci. Rep.* **2016**, *6*, 35710, doi:10.1038/srep35710.

5. Johnson, K.A.; Goody, R.S. The Original Michaelis Constant: Translation of the 1913 Michaelis–Menten Paper. *Biochemistry* **2011**, *50*, 8264–8269, doi:10.1021/bi201284u.
6. Torretta, S.; Colombo, G.; Travelli, C.; Boumya, S.; Lim, D.; Genazzani, A.A.; Grolla, A.A. The Cytokine Nicotinamide Phosphoribosyltransferase (ENAMPT.; PBEF.; Visfatin) Acts as a Natural Antagonist of C-C Chemokine Receptor Type 5 (CCR5). *Cells* **2020**, *9*, 496, doi:10.3390/cells9020496.
7. Morgan, C.A.; Hurley, T.D. Development of a High-Throughput in Vitro Assay to Identify Selective Inhibitors for Human ALDH1A1. *Chem. Biol. Interact.* **2015**, *234*, 29–37, doi:10.1016/j.cbi.2014.10.028.
8. Chen, Y.; Zhu, J.-Y.; Hong, K.H.; Mikles, D.C.; Georg, G.I.; Goldstein, A.S.; Amory, J.K.; Schönbrunn, E. Structural Basis of ALDH1A2 Inhibition by Irreversible and Reversible Small Molecule Inhibitors. *ACS Chem. Biol.* **2018**, *13*, 582–590, doi:10.1021/acschembio.7b00685.
